# Understanding the Motives of Undertaking Physical Activity with Different Levels of Intensity among Adolescents: Results of the INDARES Study

**DOI:** 10.1155/2018/1849715

**Published:** 2018-09-30

**Authors:** Magdalena Król-Zielińska, Dorota Groffik, Michał Bronikowski, Adam Kantanista, Ida Laudańska-Krzemińska, Małgorzata Bronikowska, Agata Korcz, Joanna Borowiec, Karel Frömel

**Affiliations:** ^1^Faculty of Physical Education, Sport and Rehabilitation, Poznan University of Physical Education, Królowej Jadwigi 27/39, 61-871 Poznań, Poland; ^2^Faculty of Physical Education, The Jerzy Kukuczka Academy of Physical Education in Katowice, Mikołowska 72a, 40-065 Katowice, Poland; ^3^Faculty of Tourism and Recreation, Poznan University of Physical Education, Królowej Jadwigi 27/39, 61-871 Poznań, Poland; ^4^Faculty of Physical Culture, Palacký University Olomouc, Křížkovského 511/8, CZ-771 47 Olomouc, Czech Republic

## Abstract

**Background:**

The aim of this study was to evaluate the relationship between the motives for undertaking physical activity (PA) and the intensity of PA in Polish adolescents.

**Methods:**

The study included 1,231 students, 515 boys (age 16.2 ± 0.7 years) and 716 girls (age 16.3 ± 0.6 years). The participants were recruited from secondary schools in 20 conurbations throughout Poland. The International Physical Activity Questionnaire-Long Form and the Motives for Physical Activity Measure-Revised were used.

**Results:**

In boys, all motives predicted a 10.4% variance in vigorous intensity of PA (*F*(5, 509) = 11.822,* p* < .001). Higher scores on competence and appearance motives for PA were found to be predictors of higher level of vigorous intensity of PA. In girls, all motives explained a 7.4% variance in vigorous intensity of PA (*F*(5, 710) = 11.292,* p* < .001). Higher scores on competence and appearance motives for PA were found to be predictors of higher level of vigorous intensity of PA.

**Conclusions:**

This study shows that competence and appearance related motives for PA are important motivations for Polish adolescent girls and boys in undertaking vigorous intensity PA.

## 1. Introduction

Adolescence is a crucial period in one's live. Both healthy and health-endangering habits and patterns are established during these formative years. Such habits and patterns have the potential to influence one's behavior and health status, as they mature into adulthood [1]. This issue specifically concerns habits of physical activity (PA). Regular PA reduces the risk of many diseases in youth [2, 3] and provides positive health benefits [1]. Conversely, being physically inactive directly connected to multiple negative health consequences [4]. Vigorous intensity of PA may be more beneficial than lower intensity activity in the control of one's weight [5]. It is significant to note that the vigorous intensity of PA by adolescents decreases as they age [6, 7].

Studies show a decline in PA levels in 9-18-year-olds and have indicated that the steepest decline is found between ages 15 and 18, with boys being more active than girls [8, 9]. In this context, PA practices depend primarily on one's awareness, abilities, and willingness to be physically active. Individual motivation enables adolescents to initiate and maintain regular PA which closely parallels individual interests and physical capabilities.

Motivation, as a psychological factor, stimulates an organism to act toward a preferred aim and induces, controls, and sustains certain goal-directed behaviors. Adolescents present various kinds of motivation and differ not only in the levels (i.e., how much) but also in the orientation of motivation (i.e., the underlying attitudes or intentions). The types of sports motivation, provided by Deci and Ryan [10] within self-determination theory (SDT), constitute a basic framework of motivation regulations. These types of motivations range from the specific behavior of intrinsic, to the extrinsic or demotivational types, who may be self-determined (autonomous), controlled, or due to lack of motivation. However, there are other mediating factors that need to be considered. In a systematic review on SDT, based on exercise motivation studies from 1960 through 2011, a positive relationship was identified for individuals with self-determination and their adoption and maintenance of PA [11].

Adolescent motivation in undertaking PA is related to several factors. Social-environmental factors, such as the content of the physical education classes (i.e., different activities), and meaningful adult encouragement (i.e., parents, athletes/celebrities as role-models, and cultural values) are significant in motivating adolescents to undertake PA [12, 13]. Moreover, as demonstrated by Wold et al. [14], the reasons of enjoying leisure time PA in adolescents have changed over the past 20 years.

Some analysis of sports motivation in adolescents points to the fact that boys tend to undertake more vigorous forms of sports involving competition [15] and value achievement factors in PA [16, 17]. While girls tend to undertake less intense forms of PA, they, nonetheless, pursue their PA from motivations such as fitness, well-being, and physical appearance [15]. In addition, for girls, health and social motivation are important factors when considering undertaking PA [16–18]. Another study [19] revealed the cause-effect relationship of perceived sports competences and motor skills proficiency with the level of PA and fitness in adolescents. In addition, according to Jaakkola et al. [20], perceived physical competence toward PA in adolescents was the only predictor of later PA engagement.

Limited research has been conducted on the relationship between motivation and the intensity of PA. In a study by Sibley et al. [21], the appearance motive had a negative correlation with fitness test results, unlike fitness and competence motives, that had a significantly positive relationship.

Investigating the relationships between motivation factors and intensity levels of PA in adolescents may help in better understanding the mechanism behind the engagement of adolescents in PA and improve the understanding of the mediating components that are essential to achieve higher engagement in PA. Based on the above findings, a study has been designed with the objective of evaluating the relationship between motives and intensity of PA in Polish adolescents, separately for boys and girls. It was hypothesized that a vigorous intensity of PA is associated with different motives than a moderate or low intensity of PA and that sex plays a crucial role in this relationship.

## 2. Material and Methods

### 2.1. Participants and Procedure

The study included data collected in 2015 from 1,231 students, 515 of whom were boys (age* M* = 16.2,* SD* = 0.7 years; body mass* M* = 67.0,* SD* = 12.4 kg; body height* M* = 177.3,* SD* = 7.1 cm) and 716 were girls (age* M* = 16.3,* SD* = 0.6 years; body mass* M* = 57.0,* SD* = 0.8 kg; body height* M* = 166.4,* SD* = 5.9 cm). Body height was measured to the nearest 0.5 centimeters (cm) using a stadiometer and body weight was measured to the nearest 0.1 kilogram (kg) using electronic scales (Tanita Corporation, Japan), with each participant wearing minimal clothing. Measures were taken during physical education classes and recorded by trained research assistants.

For the purpose of the study, the online system International Database for Research and Education Support (INDARES) (www.indares.com) was used [22]. The Polish version was translated from English in compliance with standardized translation guidelines, including back-translation into English. Polish version of the online system INDARES is a suitable diagnostic tool for the examination of sport and PA preferences sphere in adolescents [23]. The participants were recruited from standard urban schools, within Poland, in twenty major conurbations with more than fifty thousand (50,000) residents. The sample unit for the study was a school. Fifty-one secondary schools were randomly selected. Questionnaires were completed in whole-class groups during one regular school lesson in quiet-classroom conditions. The questionnaires were completed in approximately thirty minutes and were administrated by trained research assistants.

### 2.2. Ethics

Each of the adolescents and their parents received the study information vis-à-vis in-school meetings at which time the study information was provided and consent forms executed. Adolescents were also informed about the anonymous and voluntary nature of their participation, that the study records would be kept confidential, and that their individual contributions would be unidentified in the final report. The study was approved by the Institutional Research Ethic Committee of Palacký University Olomouc (decision no. 24/2012).

### 2.3. Physical Activity

For the objective of measuring the level of PA the International Physical Activity Questionnaire-Long Form (IPAQ-LF) was used [24]. The purpose of the questionnaire was to find out about the kinds of PA that people undertake as part of their everyday lives. The IPAQ-LF instrument has acceptable measurement properties (Spearman's *p* clustered around 0.8, criterion validity, assessed against accelerometer measures, had a median *p* of about 0.30) as for self-reports [25].

The questions ask about the time spent being physically active during the preceding seven (7) consecutive days. Physical activities were ranked as follows: moderate, activities with moderate physical effort making one breath somewhat harder than normal, and vigorous, activities with hard physical effort making one breath much harder than normal. Activities were categorized and may have been treated separately to obtain the specific activity patterns or, alternatively, multiplied by their estimated value in Metabolic Equivalent of Tasks (METs) and totaled in order to gain an overall estimate of PA in a week [26]. According to IPAQ-LF procedure, the MET intensity values used to classify the scores in the questions were vigorous (6 METs), moderate (4 METs), and low, otherwise known as walking (3.3 METs). Time spent in each activity category was derived by multiplying the number of days per week by the minutes spent doing the activity per day, while total weekly PA (MET-Min week^−1^) was calculated by multiplying the number of minutes spent in each activity category by the specific MET score for each activity. Other questions collected information on the time (i.e., the number of days, average times) spent undertaking PA. The permitted average daily sum of minutes of PA and transportation was set to 600 minutes. Due to these study requirements, 156 participants were excluded. These participants reported an unrealistic estimate of each PA category. Specifically, boys increased the time estimate and the number of days in which they performed vigorous intensity PA.

### 2.4. Motives for Physical Activity

Participants' motives were assessed with the Motives for Physical Activity Measure-Revised (MPAM-R) [27]. The internal consistency of the scale was high (Cronbach's alpha above 0.87 for each subscale). The scale consists of a total of 30 items assessing five categories of reasons for activity engagement: interest/enjoyment (7 items), competence (7 items), appearance (6 items), fitness (5 items), and social (5 items). A 7-point Likert scale (1, “not at all true for me”; 7, “very true for me”) was used to rate the reason for participating in each of the items.

### 2.5. Statistical Analysis

Descriptive statistics and* t*-test were used to examine the differences in the analyzed variables for boys and girls. Pearson correlations and multiple regressions were conducted to investigate the relationships of PA with motives for PA in boys and girls. Statistical analysis was carried out using STATISTICA software (StatSoft, Inc., USA).

## 3. Results

Basic statistical characteristics of the analyzed variables are presented in Table 1, along with the significance of differences between girls and boys. Girls differed significantly from their male peers with regard to body height, weight, and BMI (*p* < .001). Boys declared a higher level of total PA (*p* < .001) and undertook more PA with vigorous (*p* < .001) and moderate intensity (*p* < .001) in comparison to girls. The differences between boys and girls in motives could be found in all motives for PA. Boys scored higher than girls on the following motives, interest, competence, social (*p* < .001), and fitness (*p* = .020), and lower on appearance (*p* < .001).

Pearson's correlation coefficients between motives and PA in girls and boys are summarized in Table 2. In boys, a significant positive correlation was documented between total PA, moderate and vigorous intensity of PA, and all motives for PA (*p* < .05), excluding the relationship between vigorous intensity of PA and social motives. Higher scores on interest and competence were associated with low intensity of PA (*p* < .05). Among girls, a higher level of total PA and higher level of vigorous and low intensity of PA were associated with a higher score on all motives for PA (*p* < .05). However, there was no statistically significant relationship between moderate intensity of PA and examined motives for PA in girls.

To test the hypothesis that PA undertaken with different intensity is related to different motives for PA in adolescent boys and girls, a multiple regression was conducted (Tables 3 and 4).

In boys (Table 3), all motives predicted a 10.4% variance in vigorous intensity of PA (*F*(5, 509) = 11.822,* p* < .001). Higher scores on competence (*p* < .001) and appearance (*p* = .008) motives for PA were found to be an indicator of higher level of vigorous intensity of PA. Although the model consisting of different motives in prediction of moderate intensity of PA was significant (*F*(5, 509) = 2.648,* p* = .022) and explained 2.5% of variance, separately none of the motives significantly predicted moderate intensity of PA. The model consisting of different motives and low intensity of PA was not significant (*F*(5, 509) = 1.535,* p* = .177).

In girls (Table 4), all motives explained a 7.4% variance in vigorous intensity of PA (*F*(5, 710) = 11.292,* p* < .001). Higher scores on competence (*p* < .001) and appearance (*p* = .038) motives for PA were found to be predictors of higher levels of vigorous intensity of PA. The models consisting of different motives in predicting moderate and low intensity of PA were found to not be significant (*F*(5, 710) = 1.521,* p* = .181 and* F*(5, 710) = 1.925,* p* = .088, respectively).

Both in girls and boys the model consisting of different motives in predicting total PA was significant (*p* < .001) and explained a 6.3% (in boys) and 4.0% (in girls) variance in total PA. In girls and boys, higher scores on competence were associated with higher levels of total PA (*p* < .05).

## 4. Discussion

In the forgoing study, the relationship between the individual motives and intensity of PA in Polish adolescents was evaluated. The study hypothesized that PA undertaken with different intensity is related to different motives in adolescent boys and girls. It was found that, in boys, higher scores on competence and appearance motives for PA were associated with the higher level of vigorous intensity of PA. Similarly, in girls, higher scores on competence and appearance motives for PA were associated with the higher level of vigorous intensity of PA.

The findings are consistent with a previous study demonstrating that high physical competencies allow for achievement of better results in the competition which was found to be an important PA motivator for boys [28]. It is noteworthy that, in the current study, competence was also an important motive for PA among girls. This result corresponds to the reports indicating that movement competency seems to be a crucial motivating factor in determining an adolescents' ability to start/restart PA participation [29]. The motives associated with the competencies that lead to success in competition may be contrary to the social motivation, which are important to achieve success in sports competition [28]. This may explain the lower importance of social motivation noticed in the presented study findings.

In the current study, in both boys and girls, a higher score on appearance motive for PA was found to be a predictor of the higher level of vigorous PA. This result may be related to the prevalence of body dissatisfaction among adolescents [30]. Likewise, in Laudańska-Krzemińska and Bronikowski's [31] study adolescent girls were extremely critical of their bodies, which led to a lower belief in their possibilities, the will to be someone else, the need to change appearance, and less frequently perceived happiness. In girls, the desire to be thin and feminine leads to increased motivation to be physically active [32], whereas in the case of boys, low body satisfaction often leads to undertaking PA to achieve muscular body shape [33]. This finding may be associated with pressure from the mass media. Sociocultural and biological patterns of masculine and feminine body types may be the reasons that the appearance motive for PA was a predictor of the higher level of vigorous PA in the present study. Similar results have been registered in a previous study [34].

In the present study, social motivation was more important for girls than for boys, which is also in line with the results obtained by Foran et al. [35]. The importance of social motivation for girls was also registered in other studies [16, 17]. Additionally, a study by Ianotti et al. [18] showed that girls had lower social and achievement motivation, but higher health motivation than boys. Litt et al. [17] suggested that it is important to communicate the health benefits of PA as the key motive for affecting the level of PA in adolescents. This corresponds to Kalman et al.'s [16] results, which found that health is a more important value consideration for older adolescent girls, whereas Verkooijen et al. [28] indicated that health and enjoyment were similarly important for both genders. Furthermore, in our study boys reported higher rates of achievement motivation than girls. This is consistent with other studies that found achievement was more important for boys than girls [16, 18].

Our results show that, in comparison to girls, boys demonstrated a higher level of total PA and undertook more PA with vigorous and moderate intensity. A larger number of female respondents presented a low level of PA in comparison to boys. The findings of the current study are in line with the general findings of the epidemiology of PA in adolescents, where boys were more active than girls [36]. Similar results were obtained by Iannotti et al. [18], where female adolescents were less active than males, which further emphasized the role of motivation, and the different reasons for undertaking PA by adolescents. For example, for European girls and boys, achievement motivation was positively related to PA. Moreover, for girls, social motivation was also a strong predictor of PA [18].

In the present study, we found that boys scored higher than girls on the following motives, interest, competence, social, and fitness, and lower on appearance. It should be considered, that adolescents' PA is determined by a complex array of intrapersonal, interpersonal, family, school, and community environmental factors [37, 38]. Thus, the present findings may be related to extraneous factors not evaluated in the current research. For girls, especially, the motives for undertaking PA in the present study were mainly appearance-oriented. This appearance factor may be linked to the specific choice of the form of PA. Frömel et al. [39] have pointed out that girls most often prefer forms of PA dominated by smooth, aesthetic movements. Camacho-Miñano et al. [40] have recommended making PA enjoyable for girls by increasing the choices and offering a wide range of noncompetitive and innovative activities. Barr-Anderson et al. [41] have also reported that enjoyment was the key PA motivator for girls.

In consideration of the SDT, a review of forty-six studies of the association between the motivations and PA in both children and adolescents indicated that autonomous motivation had a moderate positive association with PA; conversely, controlled forms of motivation had a weak negative association with PA [42]. It appears that autonomous motivation is important mainly for adolescents. Adolescence is a crucial time of increasing autonomy. Accordingly, providing best choices and involving the young people, themselves, in designing PA programs may be worthwhile in establishing and encouraging self-initiated behavior change [43].

Predicated upon this study, and previous studies, possible strategies to improve motives of undertaking PA in adolescents should be driven (for both boys and girls) by primarily focusing on competences and appearance, especially competences that have been shown to be the crucial predictor of continued physical activity in adulthood [20]. Additionally, other areas which might strengthen the motivation should be developed and encouraged through better planning (social network, making arrangements to play with friends and prioritizing PA, effective time management), greater variety of PA (e.g., new and nontraditional activities, more intensive activities), social aspects, parental, teachers and peers' support, restructuring physical education environment, and implementation of health-oriented programs.

Some limitations and strengths of the current study are worth noting. Self-reported measures methods were used. As such, the subjective interpretation of questions may have influenced some of the findings. Future research may benefit from including other factors that may contribute to the explanation of the motives behind undertaking the various levels of PA intensity and related behaviors (e.g., self-efficacy, moral norms, past PA experiences, self-identity, and environmental factors).

Clearly, the strengths of the study include the large sample size of the adolescent female and male participants, recruitment throughout Poland, and the use of standard assessments at each measurement point. The academic acceptance of the applied research tool which allows for comparison of the present study results with findings of other researchers further validates the objective and findings of this study. Finally, the original nature of the present research demonstrates how adolescents are differently motivated toward PA of various intensities.

## 5. Conclusions

The academic research presented in this study has had as its objective the presentation of empirical data to advance the understanding of the associations between the motives and intensity of PA in Polish adolescents. The findings from the present study confirm that both boy's and girl's competence and appearance motives for PA were predictors of higher levels of vigorous intensity of PA. School physical education programs and PA interventions would be well-served to incorporate this study's findings to form a more complete understanding of the complex motivators of adolescence decision-making as it relates to vigorous PA.

Further analysis as to why competence and appearance were the main predictors of vigorous PA among young people is required. Disturbing is the fact that the study's findings express what little importance young people place on the health and social aspects of PA. Therefore, this study expresses the need to increase the awareness of young people of the physical and emotional benefits of vigorous PA. Moreover, this study points to the need for improved programs for the presentation of the best approaches for implementing and expanding the knowledge of young people on the life-long health advantages of proper PA.

In conclusion, a better understanding of the motives for PA, as well as differences between young boys and girls, can be applied as a framework to improve physical education curriculums.

In turn, this awareness may act to encourage young people to have more active lifestyles. Notwithstanding this present study, more research and analysis will continue to be necessary to add to the knowledge base to more completely understand the complex relationships driving the motivation for PA, and the intensity of PA, in adolescents.

## Figures and Tables

**Table 1:** Descriptive statistics analyzed variables and differences between boys and girls.

	Overall N = 1231	Boys n = 515	Girls n = 716	*p*
	*M* ± *SD*	*M* ± *SD*	*M* ± *SD*	
Anthropometric parameters				
Weight (kg)	61.2 ± 11.7	67.1 ± 12.4	57.0 ± 8.9	< .001
Height (cm)	171.0 ± 8.5	177.4 ± 7.2	166.4 ± 6.0	< .001
BMI (kg/m^2^)	20.9 ± 3.1	21.3 ± 3.4	20.6 ±2.8	< .001
Physical activity (MET-Min week^−1^)				
Vigorous intensity	1735.7 ± 2112.6	2053.1 ± 2228.2	1507.4 ± 1995.9	< .001
Moderate intensity	2216.0 ± 2369.9	2549.0 ± 2574.2	1976.5 ± 2182.0	< .001
Low intensity	2428.2 ± 2254.6	2307.5 ± 2238.7	2515.1 ± 2263.6	.111
Total	6379.9 ± 4839.2	6909.7 ± 5104.0	5998.9 ± 4605.6	< .001
Motives for physical activity				
Interest (pts)	35.4 ± 9.5	37.0 ± 8.9	34.3 ± 9.8	< .001
Competence (pts)	34.9 ± 9.6	37.1 ± 9.0	33.3 ± 9.7	< .001
Appearance (pts)	31.6 ± 8.7	30.0 ± 9.2	32.7 ± 8.1	< .001
Fitness (pts)	26.2 ± 6.3	26.7 ± 6.3	25.9 ± 6.2	.020
Social (pts)	20.8 ± 7.4	22.2 ± 7.4	19.8 ± 7.3	< .001
Total (pts)	148.9 ± 32.9	153.0 ± 32.9	146.0 ± 32.6	< .001

**Table 2:** Pearson's correlation coefficients between motives and PA.

Physical activity	Motives for physical activity					
	Interest	Competence	Appearance	Fitness	Social	Total
	Boys					

Vigorous intensity	.23^*∗*^	.28^*∗*^	.20^*∗*^	.18^*∗*^	.06	.24^*∗*^
Moderate intensity	.10^*∗*^	.13^*∗*^	.12^*∗*^	.15^*∗*^	.09^*∗*^	.14^*∗*^
Low intensity	.11^*∗*^	.11^*∗*^	.07	.07	.05	.10^*∗*^
Total	.20^*∗*^	.23^*∗*^	.18^*∗*^	.18^*∗*^	.09^*∗*^	.22^*∗*^

	Girls					

Vigorous intensity	.23^*∗*^	.25^*∗*^	.11^*∗*^	.15^*∗*^	.13^*∗*^	.23^*∗*^
Moderate intensity	.03	.06	.05	.06	.06	.07
Low intensity	.08^*∗*^	.09^*∗*^	.08^*∗*^	.09^*∗*^	.09^*∗*^	.11^*∗*^
Total	.15^*∗*^	.18^*∗*^	.11^*∗*^	.14^*∗*^	.13^*∗*^	.19^*∗*^

Note. ^*∗*^*p* <.05

**Table 3:** Results of multiple regressions examining the association between motives and PA in boys.

Physical activity	Motives	*B*	*β*	*T*	*P*
Vigorous intensity	*F*(5,509) = 11.822, *p* < .001, Model *R*^2^ = .104				
	(Constant)	-529.160		-1.177	.240
	Interest	31.506	.125	1.557	.120
	Competence	67.977	.274	3.436	< .001
	Appearance	35.612	.147	2.684	.008
	Fitness	-41.823	-.119	-1.708	.088
	Social	-47.726	-.158	-2.923	.004
Moderate intensity	*F*(5,509) = 2.648, *p* = .022, Model *R*^2^ = .025				
	(Constant)	807.764		1.491	.136
	Interest	-19.582	-.067	-.803	.422
	Competence	21.702	.076	.910	.363
	Appearance	10.708	.038	.670	.503
	Fitness	39.441	.097	1.337	.182
	Social	12.895	.037	.655	.512
Total	*F*(5,509) = 6.820, *p* < .001, Model *R*^2^ = .063				
	(Constant)	1498.001		1.422	.155
	Interest	36.749	.064	.775	.438
	Competence	103.562	.182	2.235	.026
	Appearance	56.490	.102	1.817	.070
	Fitness	-16.495	-.020	-.288	.774
	Social	-47.173	-.068	-1.233	.218

**Table 4:** Results of multiple regressions examining the association between motives and PA in girls.

Physical activity	Motives	*B*	*β*	*T*	*P*
Vigorous intensity	*F*(5,710) = 11.292, *p* < .001, Model *R*^2^ = .074				
	(Constant)	-370.360		-1.032	.302
	Interest	15.806	.078	1.048	.295
	Competence	56.390	.275	3.693	< .001
	Appearance	21.676	.088	2.075	.038
	Fitness	-41.392	-.128	-2.151	.032
	Social	-9.161	-.034	-.733	.464
Total	*F*(5,710) = 5.874, *p* < .001, Model *R*^2^ = .040				
	(Constant)	2216.195		2.629	.009
	Interest	-17.667	-.038	-.498	.618
	Competence	91.865	.194	2.561	.011
	Appearance	43.610	.077	1.777	.076
	Fitness	-22.825	-.031	-.505	.614
	Social	24.816	.040	.845	.398

## Data Availability

All data arising from this study are contained within the manuscript and supplementary information file.

## Supplementary Materials

Supplementary Materials Supplementary materials contain raw data concerning variables examined in the study.

## References

[1] Ortega F. B., Ruiz J. R., Castillo M. J., Sjöström M. (2008). Physical fitness in childhood and adolescence: a powerful marker of health. *International Journal of Obesity*.

[2] Eisenmann J. C., Bartee R. T., Smith D. T., Welk G. J., Fu Q. (2008). Combined influence of physical activity and television viewing on the risk of overweight in US youth. *International Journal of Obesity*.

[3] Strong W. B., Malina R. M., Blimkie C. J. R. (2005). Evidence based physical activity for school-age youth. *Journal of Pediatrics*.

[4] Tremblay M. S., LeBlanc A. G., Kho M. E. (2011). Systematic review of sedentary behaviour and health indicators in school-aged children and youth. *International Journal of Behavioral Nutrition and Physical Activity*.

[5] Janssen I., Ross R. (2012). Vigorous intensity physical activity is related to the metabolic syndrome independent of the physical activity dose. *International Journal of Epidemiology*.

[6] Corder K., Sharp S. J., Atkin A. J. (2016). Age-related patterns of vigorous-intensity physical activity in youth: The International Children's Accelerometry Database. *Preventive Medicine Reports*.

[7] Katzmarzyk P. T., Lee I.-M., Martin C. K., Blair S. N. (2017). Epidemiology of Physical Activity and Exercise Training in the United States. *Progress in Cardiovascular Diseases*.

[8] Caspersen C. J., Pereira M. A., Curran K. M. (2000). Changes in physical activity patterns in the United States, by sex and cross-sectional age. *Medicine & Science in Sports & Exercise*.

[9] Currie C., Zanotti C., Morgan A. Social Determinants of Health and Well-Being Among Young People: Health Behaviour in School-Aged Children (HBSC) Study: International Report from the 2009/2010 Survey. http://www.euro.who.int/__data/assets/pdf_file/0003/163857/Social-determinants-of-health-and-well-being-among-young-people.pdf.

[10] Deci E. L., Ryan R. M. (1985). *Intrinsic Motivation and Self-Determination in Human Behavior*.

[11] Teixeira P. J., Carraça E. V., Markland D., Silva M. N., Ryan R. M. (2012). Exercise, physical activity, and self-determination theory: a systematic review. *International Journal of Behavioral Nutrition and Physical Activity*.

[12] Sanz-Arazuri E., Ponce-de-León-Elizondo A., Valdemoros-San-Emeterio M. Á. (2012). Parental predictors of physical inactivity in spanish adolescents. *Journal of Sports Science and Medicine*.

[13] Bronikowski M., Bronikowska M., Laudańska-Krzemińska I., Kantanista A., Morina B., Vehapi S. (2015). PE teacher and classmate support in level of physical activity: The role of sex and BMI status in adolescents from Kosovo. *BioMed Research International*.

[14] Wold B., Littlecott H., Tynjälä J. (2016). Changes from 1986 to 2006 in reasons for liking leisure-time physical activity among adolescents. *Scandinavian Journal of Medicine & Science in Sports*.

[15] Sukys S., Majauskiene D., Cesnaitiene V. J., Karanauskiene D. (2014). Do Parents' Exercise Habits Predict 13-18-Year-Old Adolescents' Involvement in Sport?. *Journal of Sports Science Medicine*.

[16] Kalman M., Gecková A. M., Hamřík Z., Kopčáková J., Iannotti R. J., Veselská Z. D. (2015). Motives for physical activity among adolescents in the Czech and Slovak Republics. *Central European Journal of Public Health*.

[17] Litt D. M., Iannotti R. J., Wang J. (2011). Motivations for adolescent physical activity. *Journal of Physical Activity & Health*.

[18] Iannotti R. J., Chen R., Kololo H., Petronyte G., Haug E., Roberts C. (2013). Motivations for Adolescent Participation in Leisure-Time Physical Activity: International Differences. *Journal of Physical Activity & Health*.

[19] Barnett L. M., Morgan P. J., van Beurden E., Beard J. R. (2008). Perceived sports competence mediates the relationship between childhood motor skill proficiency and adolescent physical activity and fitness: a longitudinal assessment. *International Journal of Behavioral Nutrition and Physical Activity*.

[20] Timo J., Sami Y.-P., Anthony W., Jarmo L. (2016). Perceived physical competence towards physical activity, and motivation and enjoyment in physical education as longitudinal predictors of adolescents’ self-reported physical activity. *Journal of Science and Medicine in Sport*.

[21] Sibley B. A., Hancock L., Bergman S. M. (2013). University students' exercise behavioral regulation, motives, and physical fitness. *Perceptual and Motor Skills*.

[22] Vašíčková J., Hřebíčková H., Groffik D. (2014). Gender, age and body mass differences influencing the motivation for physical activity among polish youths. *Journal of Sports Science*.

[23] Kudláček M., Frömel K., Groffik D. (2015). Gender differences in preferences of martial arts in Polish adolescents. *Archives of Budo*.

[24] IPAQ Guidelines for Data Processing and Analysis of IPAQ. https://sites.google.com/site/theipaq/home.

[25] Craig C. L., Marshall A. L., Sjöström M. (2003). International physical activity questionnaire: 12-country reliability and validity. *Medicine & Science in Sports & Exercise*.

[26] Booth M. L. (2000). Assessment of physical activity: an international perspective. *Research Quarterly for Exercise and Sport*.

[27] Ryan R. M., Frederick C. M., Lepes D., Rubio N., Sheldon K. M. (1997). Intrinsic motivation and exercise adherence. *International Journal of Sport Psychology*.

[28] Verkooijen K. T., Nielsen G. A., Kremers S. P. J. (2009). Leisure time physical activity motives and smoking in adolescence. *Psychology of Sport and Exercise*.

[29] Lubans D. R., Morgan P. J., Cliff D. P., Barnett L. M., Okely A. D. (2010). Fundamental movement skills in children and adolescents: review of associated health benefits. *Sports Medicine*.

[30] Kantanista A., Król-Zielińska M., Borowiec J., Osiński W. (2017). Is Underweight associated with more positive body image? results of a cross-sectional study in adolescent girls and boys. *Spanish Journal of Psychology*.

[31] Laudańska-Krzemińska I., Bronikowski M. (2013). Relationships among body satisfaction. life skills and physical education in polish adolescent girls. *The Global Journal of Health and Physical Education Pedagogy*.

[32] Flintoff A., Scraton S. (2001). Stepping into Active Leisure? Young Women's Perceptions of Active Lifestyles and their Experiences of School Physical Education. *Sport, Education and Society*.

[33] Eisenberg M. E., Wall M., Neumark-Sztainer D. (2012). Muscle-enhancing Behaviors Among Adolescent Girls and Boys. *Pediatrics*.

[34] Kantanista A., Osiński W., Borowiec J., Tomczak M., Król-Zielińska M. (2015). Body image, BMI, and physical activity in girls and boys aged 14-16 years. *Body Image*.

[35] Foran A. C., Cermak S. A., Spruijt-Metz D. (2013). Psychosocial determinants of participation in moderate-to-vigorous physical activity among hispanic and latina middle School-Aged Girls. *Hispanic Health Care International*.

[36] Armstrong N. (2012). Young people are fit and active - Fact or fiction?. *Journal of Sport and Health Science*.

[37] Bush P. L., MacDonald C. (2015). Promoting Physical Activity among Adolescents: Recommendations from Correlation Research. *RETOS - Neuvas Tendencias En Educacion Fisica, Deporte Y Recreacion*.

[38] Sterdt E., Liersch S., Walter U. (2014). Correlates of physical activity of children and adolescents: A systematic review of reviews. *Health Education Journal*.

[39] Frömel K., Formankova S., Sallis J. F. (2002). Physical Activity and Sport Preferences of 10 to 14-Year-Old Children: A 5-Year Prospective Study. *Acta Universitatis Palackianae Olomucensis, Gymnica*.

[40] Camacho-Miñano M. J., LaVoi N. M., Barr-Anderson D. J. (2011). Interventions to promote physical activity among young and adolescent girls: A systematic review. *Health Education Research*.

[41] Barr-Anderson D. J., Neumark-Sztainer D., Lytle L. (2008). But i like PE: Factors associated with enjoyment of physical education class in middle school girls. *Research Quarterly for Exercise and Sport*.

[42] Owen K., Smith J., Lubans D. R., Ng J. Y. Y., Lonsdale C. (2014). Self-determined motivation and physical activity in children and adolescents: A systematic review and meta-analysis. *Preventive Medicine*.

[43] Wilson D. K., Williams J., Evans A., Mixon G., Rheaume C. (2005). Brief report: A qualitative study of gender preferences and motivational factors for physical activity in underserved adolescents. *Journal of Pediatric Psychology*.

