# Supplementary material for: Understanding the Motives of Undertaking Physical Activity with Different Levels of Intensity among Adolescents: Results of the INDARES Study
Source: Biomed Res Int. 2018 Sep 30;2018:1849715. doi: 10.1155/2018/1849715 (PMC6186376; doi:10.1155/2018/1849715)
Supplement: Supplementary Materials — contain raw data concerning variables examined in the study. [file 1849715.f1.pdf]

Sex	Weight	Height	BMI	Age	Ipaq_J MET	Ipaq_T MET	Ipaq_H MET	Ipaq_R MET	Ipaq_V MET	Ipaq_M MET	Ipaq_W MET	Ipaq_S UMME T	Mpam_ Interest	Mpam_ Compet ence	Mpam_ Appear ance	Mpam_ Fitness	Mpam_ Social	Mpam_ Sum
2	56	178	17,67	17	0	33	0	0	0	0	33	33	9	13	37	11	11	81
1	61	175	19,92	16	0	0	60	0	0	60	0	60	14	17	18	16	14	79
1	54	178	17,04	15	100	0	0	0	60	40	0	100	37	46	42	35	23	183
1	75	182	22,64	17	0	0	55	66	0	55	66	121	24	25	29	20	23	121
2	53	163	19,95	17	33	60	55	0	0	115	33	148	7	7	6	5	5	30
1	70	170	24,22	15	0	165	0	0	0	0	165	165	39	17	6	5	5	72
1	50	172	16,90	15	0	165	0	0	0	0	165	165	29	34	29	24	20	136
2	48	169	16,81	15	0	60	105	0	0	165	0	165	27	30	28	22	18	125
1	63	177	20,11	17	0	33	40	100	60	80	33	173	21	20	19	16	14	90
1	65	183	19,41	17	0	0	180	0	0	180	0	180	41	29	10	25	16	121
1	54	173	18,04	16	0	0	70	120	0	190	0	190	43	43	35	32	27	180
1	55	171	18,81	15	60	60	85	0	60	145	0	205	49	49	42	35	35	210
2	83	163	31,24	16	0	132	105	0	0	105	132	237	31	26	35	22	22	136
1	67	187	19,16	16	120	0	85	33	0	205	33	238	36	42	31	30	31	170
1	84	176	27,12	16	106	33	30	73	0	110	132	242	20	22	24	21	16	103
2	49	160	19,14	17	133	33	30	73	60	110	99	269	40	41	38	29	34	182
2	56	158	22,43	17	0	165	120	0	0	120	165	285	19	10	30	17	10	86
2	56	166	20,32	17	93	99	70	33	60	70	165	295	25	22	22	21	22	112
2	49	163	18,44	16	148,5	33	30	90	90	30	181,5	301,5	25	20	33	22	9	109
1	48	175	15,67	16	0	330	0	0	0	0	330	330	7	17	6	5	5	40
1	54	173	18,04	15	0	300	0	33	0	300	33	333	36	37	26	24	18	141
1	57	172	19,27	16	0	93	125	133	60	225	66	351	42	42	36	30	30	180
1	58	177	18,51	16	0	93	125	133	60	225	66	351	48	47	36	35	35	201
1	57	170	19,72	17	100	0	125	133	120	205	33	358	42	42	37	31	26	178
2	52	171	17,78	16	133	93	0	133	120	140	99	359	49	49	42	35	35	210
2	61	175	19,92	17	133	33	70	133	120	150	99	369	49	48	42	33	32	204
2	54	160	21,09	18	232	33	85	33	60	125	198	383	46	43	30	31	18	168
2	70	183	20,90	15	0	0	390	0	0	390	0	390	28	28	24	20	20	120
1	60	178	18,94	15	133	0	125	133	120	205	66	391	49	49	39	29	35	201
1	90	170	31,14	17	231	93	30	40	0	130	264	394	28	28	24	20	20	120
2	47	165	17,26	15	133	60	70	133	120	210	66	396	33	41	42	35	5	156
1	56	182	16,91	17	73	231	70	33	0	110	297	407	36	25	12	23	30	126
1	69	175	22,53	16	82,5	198	0	132	0	0	412,5	412,5	41	44	12	17	12	126
2	48	164	17,85	16	0	0	0	412,5	0	0	412,5	412,5	37	34	39	35	28	173
1	99	175	32,33	16	133	93	95	100	120	235	66	421	27	26	24	20	26	123
2	45	165	16,53	15	0	49,5	220	156	90	220	115,5	425,5	29	30	24	20	21	124
1	55	170	19,03	16	133	93	70	133	120	210	99	429	49	49	42	35	35	210
1	64	187	18,30	15	100	93	125	133	120	265	66	451	49	49	42	35	35	210
1	63	183	18,81	18	226	0	235	0	120	275	66	461	41	40	41	30	34	186
2	56	165	20,57	16	133	33	60	240	60	340	66	466	39	40	41	24	21	165
1	69	179	21,53	16	80	346,5	45	0	0	125	346,5	471,5	12	13	15	12	9	61
1	48	160	18,75	15	133	93	125	133	120	265	99	484	39	41	30	33	27	170
1	61	167	21,87	16	0	330	60	99	0	60	429	489	49	47	31	21	35	183
2	48	160	18,75	18	165	148,5	180	0	0	180	313,5	493,5	14	14	31	16	7	82
2	55	162	20,96	17	0	99	210	198	0	210	297	507	27	25	42	25	19	138
2	55	160	21,48	17	0	198	40	270	270	40	198	508	25	16	33	14	7	95
1	74	189	20,72	16	0	99	420	0	0	420	99	519	38	41	24	33	28	164
2	56	155	23,31	17	0	0	360	160	0	520	0	520	29	32	33	27	23	144
2	56	164	20,82	16	60	132	180	148,5	0	240	280,5	520,5	18	23	37	25	15	118
2	60	180	18,52	16	0	132	260	165	0	260	297	557	45	38	23	34	26	166
2	58	158	23,23	16	33	33	30	462	0	30	528	558	32	31	35	28	25	151
1	45	168	15,94	16	40	93	310	133	60	450	66	576	37	30	29	23	23	142
1	75	175	24,49	16	0	198	120	264	0	120	462	582	29	17	13	18	25	102
1	72	182	21,74	16	450	33	40	60	390	160	33	583	30	27	37	26	25	145
1	68	159	26,90	16	0	120	30	450	450	150	0	600	31	40	34	26	27	158
2	55	169	19,26	16	0	247,5	210	149,5	0	310	297	607	33	42	42	34	24	175
2	61	173	20,38	17	0	231	70	312	60	190	363	613	39	38	21	26	16	140
2	65	170	22,49	16	240	49,5	210	120	120	450	49,5	619,5	9	14	23	12	9	67
2	91	185	26,59	16	0	198	205	220	0	425	198	623	46	30	39	27	20	162
1	63	181	19,23	16	0	165	460	0	0	460	165	625	37	28	23	27	20	135
1	60	174	19,82	16	420	0	120	99	0	540	99	639	38	39	34	31	18	160
2	60	168	21,26	17	0	33	240	396	0	240	429	669	49	49	42	35	35	210
1	82	182	24,76	16	60	600	45	0	60	645	0	705	23	23	29	23	16	114
2	53	173	17,71	16	139	148,5	0	419	0	360	346,5	706,5	27	29	35	28	11	130
2	60	170	20,76	16	378	330	0	0	180	0	528	708	41	32	34	29	26	162

2	65	169	22,76	17	173	346,5	100	99	60	180	478,5	718,5	27	22	28	24	18	119
2	62	167	22,23	17	353	66	60	240	240	380	99	719	13	9	37	10	7	76
1	60	185	17,53	16	0	0	0	720	720	0	0	720	25	25	36	35	5	126
2	69	169	24,16	16	73	396	120	132	0	160	561	721	25	24	28	20	14	111
2	48	167	17,21	17	0	231	490	0	0	490	231	721	24	27	18	18	24	111
1	55	165	20,20	16	0	429	0	300	300	0	429	729	48	49	37	33	27	194
2	46	166	16,69	17	0	495	210	33	0	210	528	738	26	28	23	20	17	114
1	73	175	23,84	17	0	225	400	120	0	580	165	745	34	36	31	25	20	146
1	75	195	19,72	17	0	247,5	502,5	0	0	502,5	247,5	750	35	25	24	19	23	126
2	64	168	22,68	16	90	660	0	0	90	0	660	750	15	20	15	10	5	65
2	47	165	17,26	16	0	0	750	0	0	750	0	750	34	42	38	31	26	171
1	68	174	22,46	16	231	231	60	231	0	60	693	753	18	15	19	20	20	92
2	61	165	22,41	16	0	0	480	291	60	480	231	771	43	42	17	30	20	152
1	57	180	17,59	16	0	0	775	0	0	775	0	775	27	34	25	26	29	141
1	63	175	20,57	16	0	453	265	73	0	725	66	791	28	28	24	21	20	121
1	63	173	21,05	16	0	792	0	0	0	0	792	792	34	32	38	32	12	148
2	60	164	22,31	17	0	132	300	360	0	660	132	792	18	20	36	19	7	100
2	45	169	15,76	15	73	525	125	73	0	565	231	796	41	31	29	32	20	153
1	68	190	18,84	17	165	247,5	90	297	0	90	709,5	799,5	26	24	32	24	17	123
2	45	155	18,73	16	0	462	110	231	0	110	693	803	33	35	42	24	7	141
2	50	162	19,05	16	0	330	420	93	0	480	363	843	38	36	32	30	16	152
2	65	170	22,49	16	0	495	0	360	360	0	495	855	42	39	12	31	17	141
1	70	177	22,34	17	0	412,5	70	396	0	70	808,5	878,5	42	46	23	34	26	171
1	54	165	19,83	15	240	99	300	264	0	540	363	903	27	22	10	18	25	102
2	52	172	17,58	17	573	0	340	0	540	340	33	913	40	32	35	26	14	147
1	83	187	23,74	17	591	165	160	0	0	520	396	916	24	26	16	14	17	97
1	75	170	25,95	17	0	660	125	133	60	825	33	918	49	49	42	35	35	210
2	58	167	20,80	15	247,5	495	180	0	0	180	742,5	922,5	38	33	27	26	29	153
2	50	159	19,78	16	547,5	99	200	82,5	0	500	429	929	40	38	34	31	19	162
1	50	168	17,72	17	0	0	570	360	360	570	0	930	49	37	42	29	14	171
2	55	165	20,20	15	264	297	375	0	0	375	561	936	38	38	35	30	26	167
2	51	170	17,65	17	231	0	30	694	60	70	825	955	48	49	42	35	33	207
2	65	169	22,76	16	606	66	295	0	210	295	462	967	26	21	29	22	22	120
2	74	170	25,61	17	198	412,5	255	120	0	375	610,5	985,5	34	41	37	28	28	168
2	55	175	17,96	16	231	399	0	360	360	300	330	990	41	23	36	31	31	162
2	59	155	24,56	15	720	285	0	0	720	120	165	1005	29	24	42	26	23	144
2	45	156	18,49	16	33	825	30	132	0	30	990	1020	44	48	16	22	5	135
1	78	182	23,55	17	100	453	400	73	60	900	66	1026	32	34	27	24	22	139
2	55	162	20,96	18	210	330	420	66	90	540	396	1026	17	20	7	6	9	59
2	49	169	17,16	16	651	165	210	0	420	210	396	1026	31	34	23	20	19	127
2	55	170	19,03	15	247,5	231	560	0	0	560	478,5	1039	31	35	38	30	21	155
2	55	160	21,48	15	495	198	160	198	0	160	891	1051	35	35	34	23	13	140
1	39	159	15,43	15	0	93	240	720	720	300	33	1053	31	30	25	20	20	126
2	43	167	15,42	17	0	198	230	630	630	230	198	1058	37	31	28	29	15	140
2	66	165	24,24	17	378	247,5	435	0	0	615	445,5	1061	20	19	38	22	14	113
2	48	163	18,07	17	0	231	0	831	360	240	462	1062	37	40	11	16	18	122
1	55	173	18,38	16	0	165	180	720	720	180	165	1065	36	41	36	30	5	148
1	62	177	19,79	17	0	808,5	260	0	0	260	808,5	1069	48	30	41	31	22	172
2	65	175	21,22	17	0	528	145	396	0	145	924	1069	36	36	35	31	28	166
2	59	174	19,49	17	765	198	110	0	360	350	363	1073	29	28	37	25	15	134
1	55	184	16,25	16	0	297	480	297	0	480	594	1074	31	29	21	28	19	128
2	53	164	19,71	16	0	594	360	120	0	480	594	1074	49	49	40	34	26	198
1	60	160	23,44	16	0	198	450	438	0	690	396	1086	23	21	14	20	15	93
1	58	183	17,32	16	0	297	0	792	0	0	1089	1089	45	44	22	26	25	162
1	69	173	23,05	16	60	667,5	380	0	60	470	577,5	1108	35	39	39	29	25	167
2	76	175	24,82	17	0	330	420	360	360	420	330	1110	34	29	33	15	24	135
1	75	179	23,41	15	980	132	0	0	900	80	132	1112	33	36	26	30	27	152
1	54	177	17,24	17	186	577,5	350	0	120	350	643,5	1114	42	40	37	32	5	156
1	98	180	30,25	17	240	330	160	396	240	160	726	1126	35	37	30	29	22	153
2	62	165	22,77	16	0	120	30	980	900	230	0	1130	45	45	42	35	21	188
2	53	161	20,45	16	552	346,5	135	99	180	375	577,5	1133	25	25	23	21	17	111
1	51	180	15,74	16	0	495	70	577,5	0	70	1073	1143	10	14	6	19	13	62
2	55	166	19,96	16	636	49,5	40	418,5	270	280	594	1144	38	37	38	22	24	159
2	43	161	16,59	17	0	33	0	1113	1080	0	66	1146	34	28	28	22	14	126
2	50	175	16,33	17	510	82,5	525	60	60	705	412,5	1178	37	33	33	28	29	160
2	57	179	17,79	17	0	990	90	99	0	90	1089	1179	29	34	34	29	16	142
2	58	168	20,55	17	100	660	100	320	60	460	660	1180	35	29	36	16	19	135
2	50	159	19,78	16	525	198	110	349,5	540	230	412,5	1183	36	36	28	28	18	146
2	49	169	17,16	16	0	594	0	594	0	0	1188	1188	30	27	23	22	17	119

2	71	173	23,72	18	360	396	240	198	360	240	594	1194	25	26	35	24	16	126
2	68	168	24,09	17	0	231	667,5	300	300	667,5	231	1199	47	43	40	34	35	199
2	51	165	18,73	16	351	264	560	33	120	560	528	1208	28	32	33	23	23	139
2	63	166	22,86	17	0	165	0	1050	1050	0	165	1215	38	38	30	29	26	161
2	60	172	20,28	16	0	495	375	360	360	375	495	1230	25	21	38	22	24	130
2	90	175	29,39	16	0	297	540	396	0	540	693	1233	31	28	31	25	20	135
2	69	172	23,32	17	462	33	80	660	0	80	1155	1235	44	38	34	26	24	166
2	67	159	26,50	17	977,5	198	0	66	240	160	841,5	1242	43	47	39	35	18	182
2	49	170	16,96	17	0	0	1260	0	0	1260	0	1260	40	42	35	29	24	170
2	50	168	17,72	16	392	0	60	813	840	260	165	1265	46	48	40	30	24	188
2	64	177	20,43	17	273	693	70	231	0	310	957	1267	23	22	39	16	5	105
2	57	168	20,20	17	0	594	330	360	360	330	594	1284	33	35	23	18	20	129
2	53	174	17,51	16	1285	0	0	0	0	1120	165	1285	48	47	21	30	19	165
2	42	151	18,42	16	180	0	1080	49,5	0	1260	49,5	1310	42	49	21	20	25	157
2	55	161	21,22	16	720	165	0	429	0	720	594	1314	41	43	13	16	22	135
2	51	161	19,68	17	0	297	1040	0	0	1040	297	1337	34	38	39	28	28	167
2	50	165	18,37	16	313	792	150	99	0	430	924	1354	37	44	35	31	20	167
2	63	175	20,57	17	412,5	495	200	247,5	0	200	1155	1355	31	29	36	22	20	138
2	57	165	20,94	15	0	990	210	156	90	210	1056	1356	20	25	33	21	10	109
2	72	166	26,13	16	1155	0	70	132	0	70	1287	1357	37	25	27	23	22	134
2	64	160	25,00	16	198	0	120	1050	0	840	528	1368	36	26	41	34	13	150
2	55	158	22,03	16	0	693	315	360	360	315	693	1368	38	39	34	27	22	160
2	55	164	20,45	17	660	297	280	132	0	940	429	1369	36	29	32	23	5	125
1	64	177	20,43	17	876	495	0	0	0	480	891	1371	37	44	37	35	27	180
1	59	173	19,71	16	720	300	0	360	720	660	0	1380	38	19	6	6	20	89
2	59	174	19,49	17	985,5	330	75	0	0	615	775,5	1391	9	10	30	16	5	70
1	63	178	19,88	17	0	825	570	0	0	570	825	1395	36	45	38	22	28	169
1	60	180	18,52	16	498,5	660	0	240	270	320	808,5	1399	38	42	26	31	28	165
1	74	181	22,59	17	0	213	1060	133	60	1280	66	1406	40	41	31	29	27	168
2	60	175	19,59	17	625	247,5	540	0	300	700	412,5	1413	33	32	41	32	21	159
2	46	163	17,31	17	1200	99	120	0	0	1320	99	1419	34	36	26	24	22	142
2	63	165	23,14	16	240	0	30	1155	0	270	1155	1425	22	15	40	20	17	114
1	55	177	17,56	16	228,5	337,5	140	720	180	850	396	1426	42	41	14	23	20	140
2	67	160	26,17	16	330	396	0	700	540	160	726	1426	39	37	42	29	11	158
2	63	164	23,42	18	0	924	170	360	0	530	924	1454	22	25	38	18	16	119
2	49	160	19,14	17	240	693	70	453	660	70	726	1456	38	37	38	26	21	160
2	68	168	24,09	15	1140	198	120	0	540	720	198	1458	33	36	41	29	19	158
1	45	160	17,58	17	1129	186	70	80	180	790	495	1465	35	46	23	28	17	149
2	70	178	22,09	16	0	792	180	495	0	180	1287	1467	28	21	29	19	11	108
2	53	172	17,92	17	0	1040	350	82,5	0	350	1122	1472	33	36	12	23	19	123
2	64	171	21,89	17	120	330	70	960	960	190	330	1480	27	16	33	25	12	113
2	55	171	18,81	15	420	396	240	429	0	660	825	1485	29	34	36	32	10	141
1	78	182	23,55	16	495	330	45	636	0	285	1221	1506	46	43	37	30	27	183
1	78	180	24,07	17	525	330	45	609	390	525	594	1509	30	30	28	24	24	136
2	45	165	16,53	16	779	33	555	159	420	875	231	1526	24	31	18	11	8	92
2	58	168	20,55	17	1174	99	125	133	480	325	726	1531	46	40	16	23	22	147
2	54	167	19,36	16	378	247,5	640	270	390	700	445,5	1536	39	37	42	30	23	171
2	44	160	17,19	16	874	594	0	73	0	320	1221	1541	17	16	13	9	14	69
1	52	170	17,99	16	239,5	976,5	105	232,5	300	775	478,5	1554	42	42	36	31	28	179
2	47	166	17,06	17	0	165	60	1332	720	540	297	1557	38	23	36	18	25	140
2	55	160	21,48	17	720	198	0	640	480	880	198	1558	31	34	42	31	12	150
1	71	170	24,57	16	1560	0	0	0	0	1560	0	1560	29	36	42	31	12	150
2	44	160	17,19	17	1560	0	0	0	1560	0	0	1560	25	22	21	17	18	103
1	57	170	19,72	16	576	412,5	180	396	270	420	874,5	1565	27	20	12	19	7	85
1	55	170	19,03	16	0	924	180	462	0	180	1386	1566	33	25	12	20	21	111
1	78	177	24,90	17	731	330	505	0	300	705	561	1566	42	43	19	32	22	158
2	61	161	23,53	17	0	1040	375	165	0	375	1205	1580	35	33	11	24	10	113
2	50	162	19,05	16	1122	247,5	180	33	420	420	742,5	1583	31	26	42	25	12	136
2	49	161	18,90	17	0	165	510	912	0	630	957	1587	37	28	30	25	24	144
2	60	170	20,76	15	693	0	160	750	750	160	693	1603	48	46	37	35	33	199
1	85	183	25,38	15	753	93	690	73	720	790	99	1609	21	15	36	31	15	118
1	72	180	22,22	17	240	396	975	0	240	975	396	1611	14	22	9	12	7	64
2	56	179	17,48	17	714	577,5	0	320	0	440	1172	1612	21	17	36	17	25	116
2	57	164	21,19	16	133	1233	125	133	120	1405	99	1624	47	48	40	30	24	189
2	53	165	19,47	17	1508	0	130	0	600	130	907,5	1638	15	12	23	32	5	87
2	63	165	23,14	15	1188	450	0	0	0	450	1188	1638	30	26	34	24	12	126
2	59	163	22,21	16	618	715,5	235	82,5	300	955	396	1651	26	25	33	25	17	126
2	57	166	20,69	16	711	462	285	198	120	645	891	1656	28	29	42	26	22	147
1	55	181	16,79	16	511	231	875	40	0	1195	462	1657	30	37	26	23	22	138

1	80	190	22,16	15	40	0	1620	0	0	1660	0	1660	21	34	24	22	21	122
2	49	157	19,88	17	0	165	0	1500	900	600	165	1665	41	42	32	33	24	172
2	58	167	20,80	15	600	0	0	1080	1440	240	0	1680	37	33	39	22	22	153
2	68	168	24,09	17	0	1320	0	363	0	0	1683	1683	28	34	33	20	21	136
2	46	169	16,11	15	1700	0	0	0	0	1700	0	1700	26	27	25	23	18	119
2	51	162	19,43	17	429	330	90	852	720	90	891	1701	25	32	37	26	20	140
2	63	170	21,80	17	833	462	120	297	600	320	792	1712	21	26	29	22	6	104
1	76	182	22,94	17	666	976,5	75	0	0	1305	412,5	1718	22	24	34	28	21	129
1	65	183	19,41	16	0	264	1455	0	0	1455	264	1719	35	36	36	28	25	160
2	49	160	19,14	16	1272	300	0	150	960	300	462	1722	28	27	35	24	16	130
1	60	182	18,11	16	900	0	825	0	900	825	0	1725	34	46	38	34	20	172
2	52	161	20,06	16	967,5	99	570	99	300	990	445,5	1736	29	30	34	27	17	137
2	63	172	21,30	15	960	222	170	390	0	1280	462	1742	42	40	38	27	32	179
2	63	168	22,32	17	0	495	1260	0	0	1260	495	1755	28	28	25	20	22	123
2	45	164	16,73	16	495	219	330	720	720	450	594	1764	34	33	28	24	29	148
2	48	162	18,29	16	1187	577,5	0	0	840	0	924	1764	31	34	27	23	22	137
2	49	164	18,22	16	1169	519	45	33	0	545	1221	1766	34	34	29	25	25	147
2	45	160	17,58	15	533	66	420	750	1050	620	99	1769	38	36	35	33	18	160
2	51	165	18,73	17	756	396	360	264	0	720	1056	1776	43	39	40	29	28	179
1	80	187	22,88	17	1080	198	240	264	1080	240	462	1782	43	44	26	27	24	164
2	54	164	20,08	16	1782	0	0	0	1650	0	132	1782	9	15	31	35	9	99
2	60	170	20,76	17	465	1188	30	99	0	330	1452	1782	20	23	26	21	14	104
2	68	172	22,99	17	798	66	120	802,5	1080	360	346,5	1787	45	45	36	31	25	182
2	59	171	20,18	17	0	693	680	420	0	1100	693	1793	22	23	9	15	14	83
2	62	165	22,77	17	73	33	70	1617	0	110	1683	1793	28	24	27	20	17	116
1	75	183	22,40	17	660	99	1035	0	540	1155	99	1794	39	46	33	31	29	178
2	60	171	20,52	16	796,5	231	0	771	990	0	808,5	1799	39	39	38	28	17	161
2	65	164	24,17	17	902,5	495	245	165	0	405	1403	1808	35	30	40	29	25	159
2	57	158	22,83	17	633	0	120	1059	960	720	132	1812	35	36	39	33	25	168
2	51	167	18,29	16	247,5	1041	140	384	120	950	742,5	1813	11	11	6	5	13	46
1	63	170	21,80	17	0	1188	510	132	0	510	1320	1830	33	32	38	29	20	152
1	49	164	18,22	16	699	66	0	1080	1200	480	165	1845	41	43	42	30	14	170
1	53	172	17,92	16	180	1335	0	345	360	180	1320	1860	28	26	35	19	15	123
1	60	175	19,59	15	0	1188	180	495	0	180	1683	1863	34	32	36	24	27	153
2	60	161	23,15	15	480	165	1080	148,5	0	1560	313,5	1874	12	22	33	23	6	96
2	48	160	18,75	17	0	198	60	1620	1620	60	198	1878	49	41	36	31	24	181
2	45	165	16,53	17	330	660	30	882	360	90	1452	1902	49	44	28	28	23	172
2	75	168	26,57	17	706,5	198	300	698	0	1160	742,5	1903	40	42	34	21	21	158
2	57	164	21,19	16	753	132	985	33	0	1705	198	1903	23	22	25	20	14	104
2	60	158	24,03	17	372	396	330	808,5	0	570	1337	1907	28	25	37	29	17	136
2	58	165	21,30	16	560	462	870	33	0	1430	495	1925	29	25	36	26	25	141
1	81	178	25,56	16	1200	495	240	0	0	1440	495	1935	13	25	38	22	15	113
2	47	160	18,36	18	0	1155	730	60	0	790	1155	1945	28	28	19	23	20	118
2	70	172	23,66	16	330	330	1155	132	0	1155	792	1947	30	31	37	25	13	136
1	68	175	22,20	18	73	1188	0	693	0	40	1914	1954	18	18	9	16	12	73
2	50	157	20,28	16	912	198	450	396	120	450	1386	1956	26	32	34	26	21	139
2	55	165	20,20	16	133	1188	540	99	60	580	1320	1960	16	19	39	24	15	113
2	79	181	24,11	17	247,5	693	150	876	0	630	1337	1967	37	34	38	26	35	170
1	56	179	17,48	16	0	1782	95	93	60	95	1815	1970	40	42	27	30	26	165
2	55	158	22,03	17	0	1343	630	0	0	1230	742,5	1973	33	33	39	30	24	159
2	58	166	21,05	16	1153	462	360	0	1080	400	495	1975	7	7	6	5	5	30
2	51	160	19,92	17	717	0	540	720	1140	540	297	1977	27	24	33	25	16	125
1	60	171	20,52	17	975	495	280	231	0	760	1221	1981	48	46	42	35	28	199
1	46	170	15,92	16	1200	66	480	240	0	1920	66	1986	30	22	14	21	29	116
1	60	172	20,28	16	480	720	315	480	0	1995	0	1995	34	31	24	23	24	136
2	70	173	23,39	17	412,5	247,5	630	705	0	1170	825	1995	47	32	42	33	27	181
2	60	165	22,04	16	607,5	958,5	330	109,5	360	540	1106	2006	39	41	40	33	26	179
2	65	170	22,49	16	720	849	210	231	0	1350	660	2010	30	33	31	26	28	148
2	56	160	21,88	16	1190	297	90	459	0	650	1386	2036	26	28	38	18	8	118
2	53	161	20,45	16	1393	330	280	33	420	560	1056	2036	39	39	28	31	24	161
2	52	165	19,10	16	1320	33	690	0	0	2010	33	2043	43	47	37	32	29	188
1	45	170	15,57	15	0	1113	210	733	420	1570	66	2056	46	43	42	34	33	198
2	62	171	21,20	16	396	480	135	1053	360	615	1089	2064	29	19	26	20	9	103
2	49	164	18,22	17	0	495	210	1377	1080	210	792	2082	37	35	39	27	11	149
2	57	158	22,83	16	1403	330	210	148,5	0	210	1881	2091	43	32	26	29	16	146
1	70	175	22,86	17	630	1110	360	0	630	810	660	2100	31	28	26	21	20	126
2	75	177	23,94	15	120	462	0	1529	720	120	1271	2111	44	38	40	27	24	173
2	67	172	22,65	17	774	198	540	600	420	900	792	2112	47	43	39	34	32	195
2	60	162	22,86	17	665	247,5	625	577,5	300	825	990	2115	16	16	36	22	15	105

2	54	164	20,08	17	120	693	135	1188	0	255	1881	2136	18	15	16	13	11	73
1	41	160	16,02	16	1386	148,5	620	0	0	620	1535	2155	17	18	10	11	10	66
2	62	175	20,24	17	0	1617	0	544,5	0	0	2162	2162	11	15	27	19	10	82
2	53	168	18,78	16	1609	330	0	228,5	0	600	1568	2168	29	29	30	21	21	130
1	71	190	19,67	16	2013	93	0	73	1980	100	99	2179	49	49	42	35	35	210
1	50	166	18,14	16	480	0	60	1645	750	940	495	2185	39	32	31	24	25	151
1	65	172	21,97	17	100	1494	0	594	60	940	1188	2188	31	41	28	22	20	142
1	48	165	17,63	15	1860	99	30	200	300	1790	99	2189	35	39	22	30	14	140
1	55	178	17,36	17	1313	33	850	0	0	2130	66	2196	22	22	22	16	17	99
2	43	156	17,67	16	1980	186	30	0	1980	150	66	2196	34	39	38	18	9	138
1	67	177	21,39	16	1535	412,5	30	219	960	230	1007	2197	39	43	20	26	23	151
2	58	169	20,31	16	960	346,5	300	594	0	1260	940,5	2201	38	43	42	31	18	172
1	69	178	21,78	16	2226	0	0	0	0	2160	66	2226	14	14	12	10	10	60
2	52	165	19,10	17	1100	648	90	388,5	300	920	1007	2227	48	46	39	35	25	193
2	57	170	19,72	17	370	1491	317,5	49,5	0	1618	610,5	2228	31	31	26	17	22	127
2	44	157	17,85	16	1185	247,5	405	398	840	785	610,5	2236	33	34	9	30	13	119
2	54	158	21,63	15	1529	132	220	360	1710	300	231	2241	29	29	37	25	31	151
2	50	164	18,59	18	973	577,5	620	73	0	940	1304	2244	35	34	30	25	23	147
2	40	156	16,44	16	303	1299	110	548,5	630	1350	280,5	2261	34	33	33	28	22	150
1	65	170	22,49	16	1273	231	730	33	420	890	957	2267	49	49	42	35	35	210
1	98	177	31,28	16	1386	0	685	198	0	685	1584	2269	26	30	26	17	8	107
1	77	178	24,30	17	360	33	570	1320	1440	810	33	2283	29	34	42	26	12	143
2	65	175	21,22	17	264	1275	390	372	0	750	1551	2301	24	24	32	17	15	112
1	53	168	18,78	17	0	231	285	1808	720	465	1139	2324	46	47	33	33	30	189
1	61	165	22,41	15	1845	192	120	180	690	360	1287	2337	39	35	41	19	5	139
1	64	173	21,38	17	798	450	710	400	600	1560	198	2358	39	43	32	29	26	169
2	55	165	20,20	17	880	486	0	993	1680	580	99	2359	36	34	41	25	27	163
2	55	168	19,49	15	100	231	1105	924	60	1145	1155	2360	24	26	25	17	17	109
2	59	174	19,49	16	0	297	0	2064	1800	0	561	2361	47	34	36	28	20	165
2	53	168	18,78	16	840	693	835	0	0	1675	693	2368	41	46	42	32	9	170
1	54	170	18,69	15	0	2373	0	0	0	2340	33	2373	35	35	30	21	25	146
1	58	169	20,31	17	965	346,5	60	1038	1440	260	709,5	2410	39	42	27	28	13	149
1	58	182	17,51	16	1158	924	100	240	0	1300	1122	2422	25	27	9	12	22	95
2	60	162	22,86	15	0	33	1105	1287	0	1105	1320	2425	41	40	42	33	28	184
2	53	160	20,70	16	450	693	870	412,5	120	870	1436	2426	26	28	39	28	11	132
2	65	172	21,97	16	640	1209	0	577,5	0	1420	1007	2427	22	22	41	21	10	116
1	53	173	17,71	15	2249	0	180	0	1800	480	148,5	2429	23	29	32	19	11	114
2	58	170	20,07	16	339	924	225	946,5	360	705	1370	2435	40	29	18	13	23	123
1	42	152	18,18	16	0	594	0	1842	60	0	2376	2436	38	39	35	28	26	166
2	75	169	26,26	18	0	1386	245	819	0	965	1485	2450	17	20	34	10	30	111
2	50	168	17,72	17	1363	792	100	198	450	600	1403	2453	22	17	12	16	7	74
1	66	179	20,60	16	630	0	60	1769	1890	420	148,5	2459	33	43	40	29	21	166
2	59	169	20,66	16	420	966	630	450	420	1650	396	2466	19	15	29	19	10	92
1	70	174	23,12	16	1440	0	40	990	1440	40	990	2470	41	44	40	23	28	176
2	47	157	19,07	18	775	891	360	459	360	640	1485	2485	38	32	27	25	10	132
1	60	178	18,94	16	198	1155	455	693	0	455	2046	2501	35	32	16	23	22	128
2	62	164	23,05	17	575	1131	430	365	0	1610	891	2501	29	26	41	25	12	133
2	64	157	25,96	16	0	0	910	1608	0	1330	1188	2518	13	14	15	15	10	67
1	59	167	21,16	15	0	594	310	1617	0	310	2211	2521	39	43	35	33	22	172
2	63	173	21,05	17	0	792	1140	594	0	1140	1386	2526	46	45	39	33	30	193
2	44	159	17,40	15	0	693	1065	777	360	1185	990	2535	41	40	33	31	26	171
2	66	160	25,78	17	1434	396	430	280	840	710	990	2540	30	38	24	18	15	125
2	53	163	19,95	16	1596	396	485	66	720	965	858	2543	43	43	25	30	29	170
1	74	178	23,36	17	1328	607,5	0	618	1500	360	693	2553	41	40	30	31	31	173
1	50	170	17,30	15	1733	0	30	792	0	30	2525	2555	10	12	14	13	7	56
1	54	155	22,48	16	1188	1188	180	0	0	180	2376	2556	38	32	7	18	22	117
2	50	170	17,30	16	1620	396	0	540	2160	0	396	2556	19	21	34	27	5	106
1	59	172	19,94	16	411	247,5	90	1819	1560	430	577,5	2568	45	40	18	25	25	153
1	90	180	27,78	15	1410	468	30	662	1080	500	990	2570	30	29	26	25	19	129
2	82	163	30,86	17	1300	990	135	148,5	1200	235	1139	2574	41	32	35	23	26	157
2	66	173	22,05	17	1617	495	140	330	0	140	2442	2582	21	21	29	20	17	108
2	63	175	20,57	16	198	924	1050	418	0	1270	1320	2590	38	28	33	33	5	137
2	66	160	25,78	17	1116	0	135	1356	1440	375	792	2607	31	27	37	21	18	134
2	60	169	21,01	17	853	808,5	312,5	638	300	612,5	1700	2612	14	18	23	21	15	91
2	55	165	20,20	17	2313	49,5	260	0	2160	380	82,5	2623	32	32	35	29	21	149
2	52	172	17,58	17	1593	594	440	0	900	440	1287	2627	35	39	10	32	15	131
1	60	173	20,05	16	0	462	660	1524	1260	660	726	2646	9	12	39	19	5	84
2	57	166	20,69	16	1268	577,5	540	297	0	620	2063	2683	33	17	35	35	10	130
2	62	172	20,96	16	495	924	410	855	360	410	1914	2684	47	43	40	29	24	183

1	65	180	20,06	16	1413	693	500	82,5	630	540	1518	2688	30	27	32	27	20	136
1	64	180	19,75	15	2400	33	125	133	1740	885	66	2691	37	33	24	23	11	128
1	51	168	18,07	17	1142	907,5	645	0	480	845	1370	2695	37	40	30	24	24	155
1	65	175	21,22	17	379	924	697,5	700	420	1258	1023	2701	35	37	35	29	24	160
2	59	160	23,05	17	133	93	70	2405	1380	710	610,5	2701	37	37	40	30	24	168
1	88	186	25,44	17	540	0	660	1512	1260	660	792	2712	41	43	39	29	18	170
2	57	166	20,69	16	931	412,5	480	897	720	1060	940,5	2721	34	39	36	27	14	150
1	68	186	19,66	18	2358	66	0	300	2460	0	264	2724	37	32	38	27	29	163
2	43	158	17,22	15	810	808,5	900	212	810	980	940,5	2731	27	27	39	22	19	134
1	135	174	44,59	17	349,5	99	1110	1173	300	1590	841,5	2732	19	23	21	9	18	90
2	41	160	16,02	17	0	1386	0	1356	960	0	1782	2742	14	15	16	9	15	69
2	50	165	18,37	17	660	66	1620	396	0	1620	1122	2742	24	22	14	15	15	90
2	53	162	20,20	17	0	714	765	1272	480	885	1386	2751	11	12	40	14	20	97
1	70	173	23,39	16	1253	693	825	0	0	1385	1386	2771	27	36	23	15	16	117
2	95	168	33,66	16	780	1155	40	804	990	40	1749	2779	41	44	40	28	20	173
1	70	177	22,34	16	531	1671	150	431	420	1670	693	2783	40	40	33	29	14	156
2	78	176	25,18	17	670	0	480	1644	870	1000	924	2794	32	40	30	27	31	160
1	66	182	19,93	18	330	1782	210	477	180	210	2409	2799	39	38	16	19	14	126
1	71	186	20,52	16	1980	99	0	720	720	0	2079	2799	49	49	42	35	35	210
2	47	162	17,91	15	1314	594	720	180	180	1440	1188	2808	38	34	8	22	16	118
2	60	161	23,15	16	0	0	720	2091	0	1920	891	2811	28	35	30	21	19	133
2	47	160	18,36	17	1198	246	705	697,5	660	965	1221	2846	28	30	19	30	9	116
1	79	194	20,99	16	0	693	0	2160	2160	0	693	2853	48	49	36	31	26	190
2	58	166	21,05	17	1257	231	1253	120	630	1653	577,5	2860	16	18	32	20	11	97
1	80	193	21,48	16	183	198	720	1764	90	960	1815	2865	42	41	35	29	25	172
2	52	162	19,81	18	750	1386	270	462	420	270	2178	2868	28	24	39	25	23	139
2	54	165	19,83	17	1308	660	180	720	720	300	1848	2868	44	32	36	32	28	172
1	65	174	21,47	16	2678	33	165	0	1260	725	891	2876	36	41	34	30	28	169
2	46	161	17,75	17	1195	693	135	858	0	835	2046	2881	27	31	41	29	18	146
1	80	181	24,42	18	2022	540	325	0	1560	1145	181,5	2887	39	38	39	34	24	174
2	50	160	19,53	17	830	148,5	75	1848	300	275	2327	2902	24	21	30	20	17	112
2	63	167	22,59	17	1473	813	420	198	420	900	1584	2904	21	25	31	21	25	123
1	70	178	22,09	17	2160	198	0	558	2520	0	396	2916	21	32	15	19	5	92
1	67	183	20,01	15	100	396	240	2205	0	1060	1881	2941	35	37	35	29	28	164
1	50	168	17,72	17	0	1842	1100	0	0	1160	1782	2942	35	44	22	26	12	139
2	53	165	19,47	17	630	1386	210	720	720	510	1716	2946	43	46	38	30	13	170
1	64	189	17,92	18	2280	165	280	231	0	1240	1716	2956	46	48	25	29	24	172
2	58	170	20,07	16	670	577,5	400	1313	1200	440	1320	2960	39	36	37	27	27	166
2	60	170	20,76	17	990	660	1215	99	0	1215	1749	2964	21	14	38	23	7	103
2	56	158	22,43	18	0	1386	180	1404	810	180	1980	2970	33	31	29	21	20	134
2	54	163	20,32	17	822	495	1140	525	720	1140	1122	2982	22	20	37	24	7	110
2	55	173	18,38	17	960	742,5	120	1179	1080	1080	841,5	3002	23	26	27	13	8	97
2	65	176	20,98	17	1044	808,5	480	672	540	600	1865	3005	34	41	40	30	19	164
2	40	157	16,23	15	120	693	60	2160	2160	180	693	3033	41	29	20	23	28	141
2	60	160	23,44	16	1958	787,5	120	169,5	1200	1340	495	3035	29	31	30	22	12	124
1	55	172	18,59	16	2933	33	40	33	2340	600	99	3039	30	38	32	23	20	143
2	60	174	19,82	17	712,5	181,5	640	1506	930	1120	990	3040	48	47	42	35	32	204
2	65	165	23,88	16	1485	198	690	680	1440	1250	363	3053	47	45	42	35	34	203
1	55	174	18,17	15	1440	594	1030	0	1080	1390	594	3064	23	26	28	19	15	111
1	66	175	21,55	17	720	808,5	830	720	1440	830	808,5	3079	34	34	39	28	13	148
1	76	176	24,54	16	1440	808,5	840	0	1440	840	808,5	3089	31	37	14	30	9	121
2	55	173	18,38	17	1674	159	1225	33	1080	1285	726	3091	20	15	39	30	7	111
2	50	168	17,72	16	2430	0	90	577,5	1440	90	1568	3098	38	40	27	29	24	158
1	95	182	28,68	16	798	967,5	1020	331	420	2020	676,5	3117	31	27	25	19	27	129
1	55	172	18,59	15	1715	501	482,5	438	120	2093	924	3137	49	49	34	34	32	198
1	53	178	16,73	15	1862	333	0	960	1560	1100	495	3155	38	41	39	28	24	170
1	65	179	20,29	16	0	495	0	2664	1440	960	759	3159	44	44	23	32	29	172
2	56	161	21,60	15	379	1545	905	330	0	2235	924	3159	22	24	31	24	25	126
2	63	163	23,71	15	225	412,5	280	2256	600	280	2294	3174	49	46	38	33	31	197
2	87	178	27,46	18	0	660	0	2520	2520	0	660	3180	35	31	36	26	22	150
1	61	184	18,02	16	0	198	2790	198	0	2790	396	3186	39	30	26	26	20	141
2	57	162	21,72	17	0	445,5	510	2240	2160	590	445,5	3196	8	7	29	9	6	59
2	58	164	21,56	17	0	808,5	1080	1314	0	1800	1403	3203	15	18	35	25	8	101
1	64	177	20,43	17	1530	330	420	933	540	660	2013	3213	42	43	30	33	19	167
2	67	176	21,63	17	630	2213	340	49,5	0	2440	792	3232	37	34	42	31	24	168
1	78	182	23,55	16	3240	0	0	0	3240	0	0	3240	49	49	42	35	35	210
2	51	170	17,65	16	0	2262	187,5	802,5	540	2168	544,5	3252	45	48	30	34	22	179
1	64	173	21,38	16	0	3003	125	133	60	165	3036	3261	37	32	33	28	25	155
1	75	183	22,40	17	3033	0	0	231	2340	0	924	3264	26	22	29	22	12	111

2	66	163	24,84	16	1293	418,5	630	928,5	780	1780	709,5	3270	29	24	15	15	19	102
2	65	170	22,49	16	1380	577,5	240	1076	1080	560	1634	3274	46	46	38	32	27	189
2	60	173	20,05	16	1293	976,5	240	767,5	720	1270	1287	3277	36	26	32	22	22	138
2	68	167	24,38	15	2608	450	125	100	1860	1175	247,5	3283	27	29	24	20	20	120
2	53	166	19,23	18	0	1254	0	2046	0	0	3300	3300	28	26	23	20	21	118
2	48	156	19,72	18	0	2814	490	0	0	730	2574	3304	25	24	23	26	18	116
2	53	164	19,71	17	990	1386	310	622	0	470	2838	3308	37	32	15	22	20	126
2	43	154	18,13	16	3053	93	30	133	60	1450	1799	3309	28	28	24	20	20	120
2	52	164	19,33	16	1605	1188	280	247,5	420	640	2261	3321	16	21	29	14	11	91
2	57	168	20,20	17	1212	346,5	870	894	660	1590	1073	3323	45	47	37	35	24	188
2	60	170	20,76	17	1710	912	420	297	750	1170	1419	3339	47	32	28	28	12	147
1	51	173	17,04	18	2070	783	510	0	1080	600	1683	3363	14	12	15	15	8	64
2	62	170	21,45	17	1511	693	700	462	0	940	2426	3366	36	37	42	34	10	159
1	68	175	22,20	17	2310	495	435	132	0	435	2937	3372	46	37	37	34	22	176
2	41	155	17,07	17	2772	300	350	0	0	650	2772	3422	35	39	42	28	10	154
2	54	166	19,60	16	1950	675	60	744	1200	480	1749	3429	41	34	39	28	24	166
2	51	159	20,17	17	2275	693	315	150	150	1435	1848	3433	46	47	36	31	26	186
2	53	160	20,70	15	1663	792	0	990	750	500	2195	3445	41	41	40	33	11	166
1	63	174	20,81	16	0	2541	445	462	0	445	3003	3448	34	30	16	20	22	122
2	53	162	20,20	16	0	1733	570	1155	0	570	2888	3458	34	27	29	20	13	123
2	60	165	22,04	17	1200	577,5	900	792	0	2100	1370	3470	31	26	38	29	17	141
2	50	157	20,28	16	1760	1178	270	264	0	1590	1881	3471	38	31	34	30	24	157
1	65	170	22,49	16	745	967,5	1000	765	780	2120	577,5	3478	41	34	32	25	29	161
2	47	160	18,36	17	2595	165	720	0	2100	720	660	3480	46	42	29	28	16	161
2	56	175	18,29	16	0	3204	280	0	0	3220	264	3484	10	33	19	24	6	92
2	50	169	17,51	17	0	396	300	2802	2340	300	858	3498	45	44	40	30	28	187
2	55	161	21,22	16	2044	1380	75	0	0	2245	1254	3499	27	31	36	31	18	143
2	48	163	18,07	17	1485	462	430	1125	630	430	2442	3502	45	41	25	24	17	152
1	55	172	18,59	16	1918	231	0	1380	2700	400	429	3529	47	45	30	27	23	172
1	58	175	18,94	18	280	33	2965	253	420	3045	66	3531	33	41	31	27	20	152
2	46	160	17,97	16	1431	924	360	819	1440	840	1254	3534	32	27	29	17	24	129
1	70	186	20,23	16	2947	0	320	279	180	2920	445,5	3546	44	40	34	27	24	169
2	72	169	25,21	17	780	945	1585	240	450	2275	825	3550	26	19	33	16	19	113
2	55	170	19,03	17	2631	231	90	600	1800	1290	462	3552	44	40	38	35	16	173
2	48	164	17,85	16	0	1980	685	891	0	685	2871	3556	25	22	26	28	30	131
1	63	175	20,57	16	2408	330	120	720	2160	840	577,5	3578	21	19	40	15	15	110
2	75	167	26,89	15	1740	165	180	1500	2580	180	825	3585	30	26	37	23	5	121
1	68	170	23,53	16	0	1248	630	1716	960	1710	924	3594	31	23	11	21	10	96
2	48	167	17,21	17	600	462	880	1692	1440	1600	594	3634	45	44	34	28	24	175
2	53	170	18,34	16	0	1617	1400	630	180	1520	1947	3647	28	29	39	23	10	129
2	54	165	19,83	16	2280	990	180	198	0	1140	2508	3648	44	44	42	35	29	194
1	62	182	18,72	17	2175	1224	270	0	840	1410	1419	3669	37	36	26	35	25	159
1	63	185	18,41	17	2460	792	420	0	0	900	2772	3672	28	28	24	20	20	120
2	55	168	19,49	16	2655	297	625	99	1440	1345	891	3676	40	44	40	29	18	171
2	60	170	20,76	18	547,5	2079	270	792	300	270	3119	3689	37	39	28	24	23	151
1	52	182	15,70	16	852	1476	30	1332	1260	1770	660	3690	49	49	34	35	32	199
1	60	180	18,52	15	1443	1353	420	504	450	1620	1650	3720	46	46	39	34	33	198
2	67	166	24,31	17	1905	1188	630	0	1620	750	1353	3723	40	37	41	33	5	156
1	120	195	31,56	17	3633	0	0	100	60	3640	33	3733	41	43	36	32	27	179
2	78	158	31,24	16	0	3483	125	133	60	3615	66	3741	7	7	33	21	5	73
1	70	176	22,60	17	0	60	85	3600	0	3745	0	3745	49	49	42	29	35	204
2	54	164	20,08	17	0	3093	660	0	0	3060	693	3753	22	20	17	15	8	82
2	60	170	20,76	17	0	0	1800	1971	1080	1800	891	3771	40	48	34	22	20	164
2	57	166	20,69	15	0	1716	805	1253	0	1365	2409	3774	17	16	24	14	15	86
1	61	174	20,15	17	1080	1188	660	852	1800	660	1320	3780	39	38	37	32	23	169
2	42	163	15,81	15	1369	693	630	1093	0	1590	2195	3785	19	17	32	16	17	101
2	43	164	15,99	15	0	2277	140	1386	0	140	3663	3803	44	45	22	28	31	170
1	71	190	19,67	17	838	1386	885	720	600	1645	1584	3829	39	27	32	31	6	135
1	77	185	22,50	18	0	594	0	3240	3240	0	594	3834	25	25	40	24	12	126
2	54	163	20,32	15	1463	1298	80	996	810	1640	1386	3836	42	39	38	29	18	166
2	58	182	17,51	17	1076	330	700	1740	2010	1440	396	3846	45	40	37	35	8	165
1	50	168	17,72	16	1223	582	1515	540	1050	2315	495	3860	45	43	19	20	14	141
1	53	171	18,13	16	0	924	240	2700	2700	240	924	3864	38	40	26	24	29	157
2	62	170	21,45	16	855	808,5	1960	247,5	180	2140	1551	3871	38	38	40	34	27	177
2	49	169	17,16	16	2445	115,5	402,5	918	2520	882,5	478,5	3881	30	42	6	12	31	121
2	44	164	16,36	16	3211	612	0	60	3000	190	693	3883	33	28	35	17	11	124
1	67	183	20,01	18	2160	855	55	820	540	2855	495	3890	37	35	27	29	18	146
1	62	170	21,45	16	3393	427,5	80	0	2160	1460	280,5	3901	34	37	31	29	12	143
2	40	159	15,82	17	2528	247,5	480	684	420	780	2739	3939	38	26	29	22	15	130

1	65	185	18,99	17	3285	132	270	266	120	2150	1683	3953	32	31	8	18	22	111
2	51	165	18,73	16	2086	148,5	180	1551	1320	880	1766	3966	45	37	35	26	24	167
1	56	185	16,36	17	974	1758	30	1209	1080	1010	1881	3971	36	33	24	27	21	141
2	60	163	22,58	17	1749	1118	385	742	1470	1385	1139	3994	26	27	40	24	16	133
1	90	175	29,39	17	1183	2408	0	405	630	2260	1106	3996	29	35	28	23	23	138
2	58	167	20,80	17	2355	462	180	1000	1620	760	1617	3997	41	43	15	20	22	141
2	48	169	16,81	17	1897	33	1515	558	360	1795	1848	4003	35	35	30	25	25	150
2	59	165	21,67	17	2831	742,5	430	0	0	3030	973,5	4004	46	42	42	32	26	188
2	57	170	19,72	17	2113	1782	30	82,5	0	1730	2277	4007	9	9	11	10	9	48
2	50	155	20,81	17	0	1848	250	1911	0	1930	2079	4009	26	39	37	31	22	155
1	60	180	18,52	17	3732	0	280	0	780	2440	792	4012	29	36	32	26	21	144
2	57	174	18,83	17	471	2574	630	345	0	1050	2970	4020	39	40	41	33	25	178
2	63	173	21,05	18	2220	1617	140	60	2220	200	1617	4037	44	38	31	26	14	153
2	53	170	18,34	15	1245	660	1640	495	180	1880	1980	4040	42	36	30	29	27	164
1	83	188	23,48	17	480	696	0	2880	2160	1830	66	4056	37	31	30	20	19	137
2	60	169	21,01	16	0	1782	690	1584	0	690	3366	4056	38	38	34	26	29	165
2	42	153	17,94	17	1051	346,5	45	2628	2160	145	1766	4071	40	37	17	31	11	136
2	51	168	18,07	16	3090	495	150	336	2430	750	891	4071	34	36	34	28	22	154
2	55	175	17,96	16	1238	990	600	1260	0	1860	2228	4088	33	35	33	24	20	145
2	54	169	18,91	17	2773	1113	70	133	2760	1230	99	4089	36	29	34	25	25	149
2	45	165	16,53	16	1602	1386	0	1104	420	900	2772	4092	30	35	29	27	19	140
1	69	178	21,78	16	651	2754	230	460	600	690	2805	4095	39	26	19	17	24	125
2	70	164	26,03	17	33	3465	140	462	0	140	3960	4100	26	23	41	29	9	128
1	74	180	22,84	17	287,5	247,5	0	3573	2340	1240	528	4108	45	47	33	30	25	180
2	52	160	20,31	17	2414	577,5	540	577,5	450	540	3119	4109	42	33	35	28	28	166
1	49	172	16,56	17	1114	528	1350	1133	0	2590	1535	4125	49	45	30	35	27	186
2	55	169	19,26	16	2970	1155	0	0	0	0	4125	4125	23	23	35	18	16	115
2	54	169	18,91	16	2327	198	170	1431	1440	1910	775,5	4126	48	45	41	34	22	190
1	63	170	21,80	17	1902	297	0	1931	0	2480	1650	4130	29	22	11	26	20	108
2	60	170	20,76	16	3906	0	90	165	2520	90	1551	4161	16	14	33	13	9	85
2	52	174	17,18	16	2700	450	490	531	1800	2140	231	4171	49	49	41	35	18	192
2	50	162	19,05	17	273	1155	1205	1542	600	1925	1650	4175	38	40	20	16	19	133
2	45	164	16,73	15	0	4158	40	0	0	40	4158	4198	35	33	35	19	26	148
2	51	164	18,96	17	1129	1478	1253	340	300	2233	1667	4199	35	43	41	28	16	163
1	79	185	23,08	17	2697	148,5	0	1359	2340	0	1865	4205	37	39	25	23	17	141
2	68	168	24,09	16	2255	1617	140	198	900	340	2970	4210	27	27	32	17	19	122
2	72	170	24,91	16	3440	0	490	280	840	3370	0	4210	34	41	32	32	23	162
2	58	167	20,80	16	2475	297	1440	0	0	1440	2772	4212	30	23	39	22	19	133
1	60	174	19,82	15	0	841,5	1705	1669	720	2225	1271	4216	49	48	36	35	30	198
1	57	170	19,72	15	33	4158	0	33	0	0	4224	4224	49	48	42	35	35	209
1	55	169	19,26	15	1929	1617	0	693	0	1120	3119	4239	49	49	42	35	32	207
1	80	172	27,04	17	0	4158	0	82,5	0	0	4241	4241	42	42	6	25	27	142
1	120	178	37,87	16	758	450	2715	320	0	4045	198	4243	19	28	25	29	8	109
2	60	170	20,76	16	1540	2145	560	0	420	1680	2145	4245	24	21	41	25	22	133
2	64	165	23,51	16	2070	693	300	1194	960	1020	2277	4257	47	35	39	33	31	185
2	55	162	20,96	16	3633	594	0	33	0	3600	660	4260	12	10	36	17	6	81
1	70	183	20,90	16	0	1911	945	1451	420	2765	1122	4307	29	35	7	11	7	89
1	74	180	22,84	16	500	508,5	730	2580	2640	1530	148,5	4319	45	45	33	31	26	180
1	80	180	24,69	15	2340	660	1320	0	1440	1560	1320	4320	22	21	18	15	14	90
1	58	183	17,32	16	4320	0	0	0	4320	0	0	4320	23	27	36	35	20	141
1	61	169	21,36	16	0	1080	540	2700	2340	1980	0	4320	34	42	41	28	20	165
2	54	158	21,63	16	1258	1413	317,5	1335	1320	1238	1766	4323	48	34	37	24	21	164
1	48	168	17,01	16	1800	132	2400	0	1080	3120	132	4332	27	39	28	30	20	144
2	55	165	20,20	17	693	2697	80	880	360	1680	2310	4350	44	29	38	23	22	156
2	51	173	17,04	17	412,5	997,5	780	2160	2160	1200	990	4350	49	49	15	29	32	174
1	69	174	22,79	16	768	1782	1080	739	360	1600	2409	4369	39	40	41	29	23	172
1	90	165	33,06	16	4343	0	40	0	0	3640	742,5	4383	24	22	33	23	26	128
2	45	164	16,73	16	1830	765	510	1287	1710	1890	792	4392	46	47	36	33	25	187
1	86	179	26,84	15	0	165	1020	3219	1620	2520	264	4404	45	37	40	27	24	173
2	48	158	19,23	17	133	2640	960	675	240	1000	3168	4408	36	35	23	21	24	139
1	85	185	24,84	17	3488	231	505	198	1440	705	2277	4422	38	40	35	27	10	150
1	63	180	19,44	16	1530	247,5	370	2280	3060	790	577,5	4428	46	39	16	26	32	159
2	54	165	19,83	16	3350	462	497,5	126	420	3158	858	4436	45	41	29	35	24	174
2	55	162	20,96	16	0	3906	540	0	0	3060	1386	4446	28	25	32	16	19	120
1	48	176	15,50	16	3309	462	495	198	2160	1215	1089	4464	40	36	9	14	28	127
1	65	179	20,29	17	0	93	125	4258	60	225	4191	4476	40	44	32	33	26	175
1	65	175	21,22	15	2573	238,5	1110	558	960	2760	759	4479	37	38	36	31	30	172
1	70	193	18,79	15	2160	1155	0	1188	2160	0	2343	4503	40	34	27	26	29	156
2	56	169	19,61	17	2184	582	990	753	840	1590	2079	4509	33	36	30	22	18	139

2	56	167	20,08	16	1512	990	1260	750	750	1980	1782	4512	42	36	41	30	20	169
2	54	163	20,32	18	2007	594	1590	339	720	1830	1980	4530	38	38	37	32	27	172
2	80	161	30,86	16	3283	877,5	375	0	630	2965	940,5	4536	40	33	31	22	21	147
2	54	167	19,36	17	2173	1113	360	900	2280	2200	66	4546	22	15	38	17	17	109
1	64	184	18,90	17	2145	1617	390	396	0	390	4158	4548	46	24	15	24	31	140
2	65	168	23,03	17	1657	2358	270	279	180	2470	1914	4564	35	35	39	26	18	153
2	46	158	18,43	16	1968	2079	330	219	120	450	4026	4596	43	30	26	27	30	156
2	50	172	16,90	17	0	2310	210	2079	0	210	4389	4599	16	15	25	26	10	92
1	90	164	33,46	15	1960	2160	480	0	0	4600	0	4600	28	27	36	31	15	137
2	55	162	20,96	17	2587	1035	260	720	1530	960	2112	4602	44	41	37	29	27	178
1	58	174	19,16	15	373	2228	170	1836	300	330	3977	4607	36	40	34	28	21	159
2	75	170	25,95	17	1185	2541	420	462	900	540	3168	4608	28	38	37	23	24	150
2	68	177	21,71	16	804	1188	680	1937	1650	880	2079	4609	47	35	30	29	20	161
2	56	168	19,84	17	2107	693	1820	0	90	2220	2310	4620	44	32	23	22	25	146
2	70	165	25,71	16	1560	198	0	2868	3900	0	726	4626	35	36	41	29	23	164
2	51	165	18,73	17	0	3234	1340	73	0	1380	3267	4647	49	48	34	33	32	196
2	55	163	20,70	16	1310	792	2160	396	0	2480	2178	4658	22	27	32	20	12	113
2	78	172	26,37	16	3030	462	210	960	960	930	2772	4662	43	35	31	26	28	163
1	87	185	25,42	17	0	2037	1240	1386	0	1660	3003	4663	30	28	24	22	20	124
2	62	168	21,97	17	3449	577,5	440	198	2520	560	1584	4664	29	28	21	18	25	121
2	69	161	26,62	17	0	297	410	3967	3450	630	594	4674	35	20	40	25	11	131
2	65	167	23,31	16	3765	33	410	491	2220	2050	429	4699	24	24	21	20	17	106
2	45	160	17,58	16	0	4158	570	0	0	570	4158	4728	20	29	41	32	18	140
2	59	167	21,16	16	2600	462	280	1400	840	3440	462	4742	42	34	42	27	25	170
2	69	170	23,88	17	2010	396	360	1980	810	1560	2376	4746	28	28	29	22	16	123
1	50	176	16,14	15	0	4218	540	0	0	600	4158	4758	39	41	35	31	30	176
1	65	182	19,62	16	1830	2832	0	99	840	60	3861	4761	14	20	26	12	5	77
1	79	180	24,38	17	0	1413	1470	1902	1440	2190	1155	4785	36	37	35	28	30	166
1	75	180	23,15	16	3737	675	245	132	2880	985	924	4789	46	49	40	32	26	193
2	59	174	19,49	16	2737	1238	700	126	60	1820	2921	4801	28	16	17	20	7	88
2	76	177	24,26	16	0	1617	2310	887,5	270	2350	2195	4815	27	32	42	28	14	143
2	48	158	19,23	16	450	1964	90	2326	450	370	4010	4830	27	21	25	18	16	107
2	40	160	15,63	16	0	3465	270	1118	540	270	4043	4853	42	35	37	29	25	168
2	50	166	18,14	17	1809	937,5	930	1182	1530	2190	1139	4859	31	35	32	31	21	150
1	78	183	23,29	17	1995	1617	360	891	900	960	3003	4863	37	34	26	26	27	150
2	62	167	22,23	17	1620	990	1055	1198	630	2055	2178	4863	44	37	26	21	24	152
2	53	170	18,34	18	1080	2079	220	1485	0	1300	3564	4864	34	40	30	26	22	152
1	57	176	18,40	17	0	2970	720	1188	0	720	4158	4878	30	29	32	23	18	132
2	48	152	20,78	16	1956	594	955	1377	2160	1435	1287	4882	43	42	41	28	19	173
1	52	175	16,98	17	412,5	1395	2385	720	720	3285	907,5	4913	43	43	21	34	29	170
1	75	185	21,91	16	0	462	420	4032	3240	420	1254	4914	35	42	21	27	17	142
2	68	180	20,99	16	3670	577,5	90	577,5	0	790	4125	4915	29	34	42	27	21	153
1	60	179	18,73	16	607,5	247,5	690	3372	3600	690	627	4917	39	33	14	19	22	127
1	78	176	25,18	16	0	1386	480	3062	2520	560	1848	4928	38	36	37	27	25	163
2	52	168	18,42	17	1155	594	90	3096	2700	90	2145	4935	45	46	42	31	10	174
2	51	165	18,73	16	1995	396	1470	1081	0	2830	2112	4942	30	29	27	22	18	126
1	74	170	25,61	17	4353	594	0	0	4320	0	627	4947	43	45	24	22	26	160
1	86	180	26,54	17	810	930	335	2889	3360	1175	429	4964	49	49	38	35	21	192
1	62	189	17,36	18	4932	33	0	0	0	2160	2805	4965	41	43	35	28	26	173
2	58	163	21,83	17	3600	594	180	630	630	3780	594	5004	37	42	42	23	5	149
1	67	174	22,13	15	2394	247,5	820	1554	1800	1780	1436	5016	46	39	27	33	28	173
1	55	171	18,81	16	4463	330	30	198	450	3630	940,5	5021	36	36	11	21	6	110
1	69	179	21,53	17	3913	330	705	73	3600	1025	396	5021	47	49	39	29	16	180
1	55	177	17,56	16	1786	3003	220	33	360	260	4422	5042	43	38	21	28	27	157
1	56	178	17,67	15	2751	219	595	1491	3780	715	561	5056	40	45	36	35	15	171
1	58	175	18,94	16	4299	165	595	0	1800	2995	264	5059	37	31	18	26	24	136
2	59	175	19,27	16	495	1386	420	2760	2160	1020	1881	5061	36	34	39	30	13	152
2	64	176	20,66	16	0	1733	0	3353	1620	0	3465	5085	41	44	41	30	19	175
2	50	165	18,37	17	330	693	1195	2880	3090	1315	693	5098	26	29	35	25	16	131
1	63	177	20,11	17	3036	1607	60	396	450	2520	2129	5099	33	40	38	30	24	165
2	50	173	16,71	16	3780	495	420	417	1800	540	2772	5112	27	13	24	26	21	111
1	64	170	22,15	16	1800	615	55	2646	1080	2485	1551	5116	30	26	39	22	17	134
2	80	173	26,73	17	247,5	3312	180	1386	0	2700	2426	5126	38	37	39	31	22	167
2	54	165	19,83	15	3558	0	1573	0	2100	2173	858	5131	19	15	36	15	8	93
2	43	165	15,79	16	619	2718	1200	594	240	4000	891	5131	46	48	16	33	21	164
1	60	180	18,52	16	3735	132	1170	99	3240	1170	726	5136	28	38	25	25	9	125
1	63	175	20,57	15	4755	330	70	0	0	3670	1485	5155	21	14	6	5	5	51
1	60	175	19,59	16	2590	330	350	1902	2400	990	1782	5172	37	30	13	23	20	123
2	62	173	20,72	17	1097	2331	490	1260	1890	2710	577,5	5178	47	49	26	34	17	173

2	57	168	20,20	16	1560	990	570	2108	3180	810	1238	5228	39	36	40	32	22	169
2	56	173	18,71	17	93	1103	585	3448	2220	1045	1964	5229	35	40	40	25	21	161
1	90	187	25,74	17	4446	66	330	393	2160	1590	1485	5235	41	37	22	26	15	141
2	57	170	19,72	18	0	4158	1080	0	0	1080	4158	5238	43	46	40	33	32	194
2	51	167	18,29	16	4470	0	750	33	0	2250	3003	5253	30	34	36	28	17	145
1	78	179	24,34	16	2278	2175	360	445	300	3720	1238	5258	44	28	19	29	28	148
2	55	168	19,49	17	3479	693	630	462	0	2030	3234	5264	17	19	16	19	13	84
2	53	164	19,71	15	4620	366	190	99	990	1150	3135	5275	36	35	35	28	24	158
2	70	165	25,71	17	4158	627	0	495	0	0	5280	5280	43	40	41	31	23	178
2	52	166	18,87	16	3463	492	175	1151	120	1795	3366	5281	47	48	30	35	31	191
1	58	172	19,61	16	4330	33	490	429	1080	770	3432	5282	32	45	26	24	11	138
2	66	179	20,60	17	3874	1188	0	231	2160	1120	2013	5293	34	43	28	23	12	140
1	60	167	21,51	18	762,5	2145	250	2145	270	330	4703	5303	47	49	35	35	23	189
1	75	188	21,22	17	3004	99	1045	1179	1080	3125	1122	5327	34	36	27	17	13	127
2	50	160	19,53	16	2145	1386	300	1508	1260	300	3779	5339	34	27	42	24	5	132
2	53	177	16,92	16	1410	612	1800	1518	1740	2280	1320	5340	44	49	12	29	23	157
1	90	181	27,47	17	2233	2970	100	49,5	2160	140	3053	5353	39	38	28	28	21	154
2	60	167	21,51	16	4998	0	0	360	1200	0	4158	5358	44	49	42	35	12	182
2	59	168	20,90	17	1000	1980	1660	720	1560	1820	1980	5360	36	40	23	20	22	141
1	72	185	21,04	16	525	1271	255	3312	2160	975	2228	5363	44	41	26	24	24	159
2	51	166	18,51	15	3275	297	142,5	1649	3960	462,5	940,5	5363	25	29	40	21	5	120
1	97	187	27,74	17	1530	990	270	2586	1740	270	3366	5376	39	43	42	31	19	174
2	69	165	25,34	17	1790	1386	80	2124	1200	880	3300	5380	40	34	37	32	16	159
1	59	173	19,71	16	0	924	4200	264	0	4200	1188	5388	35	30	42	26	18	151
2	60	175	19,59	17	4998	99	300	0	0	1140	4257	5397	35	27	20	25	19	126
2	57	174	18,83	17	968	3966	305	160	360	4445	594	5399	31	30	14	16	20	111
2	60	167	21,51	15	2518	1188	135	1560	2940	415	2046	5401	25	25	42	23	8	123
2	61	167	21,87	16	4157	714	540	0	2340	860	2211	5411	45	46	25	25	20	161
2	59	164	21,94	17	753	4158	210	297	0	930	4488	5418	29	29	42	29	17	146
2	59	175	19,27	16	330	1116	540	3459	2880	1740	825	5445	32	38	34	27	21	152
1	81	183	24,19	16	3177	462	1710	99	0	3270	2178	5448	25	20	39	27	12	123
1	58	178	18,31	16	3600	180	1430	240	2160	3290	0	5450	20	24	22	20	16	102
2	42	162	16,00	15	2340	1155	285	1673	2880	1005	1568	5453	32	23	8	18	5	86
1	64	183	19,11	16	2613	1386	1255	231	1080	2095	2310	5485	39	41	32	33	32	177
1	51	178	16,10	16	1113	1485	930	1980	1980	1350	2178	5508	25	29	15	16	16	101
2	53	158	21,23	17	495	1554	300	3177	2880	1260	1386	5526	42	40	31	30	22	165
1	65	176	20,98	17	4080	555	172,5	720	1440	3593	495	5528	48	44	29	26	15	162
1	55	184	16,25	16	1265	577,5	630	3075	2580	1730	1238	5548	37	45	37	31	25	175
1	63	175	20,57	17	5400	148,5	0	0	5400	0	148,5	5549	30	37	27	35	10	139
2	57	171	19,49	17	2555	1238	1770	40	0	3210	2393	5603	35	29	32	25	24	145
2	54	161	20,83	16	1172	2772	260	1428	0	1540	4092	5632	33	25	36	24	15	133
2	50	167	17,93	16	3486	1215	710	240	2100	1670	1881	5651	30	33	31	27	26	147
2	53	170	18,34	17	0	4158	345	1155	0	345	5313	5658	47	46	18	31	15	157
1	83	191	22,75	16	1932	1386	510	1836	990	2430	2244	5664	28	20	26	23	10	107
1	67	175	21,88	17	175,5	3633	1225	631	240	2075	3350	5665	29	27	37	23	6	122
1	73	184	21,56	17	1328	3030	30	1287	1080	90	4505	5675	28	37	32	20	23	140
1	74	179	23,10	16	2925	1386	240	1134	960	600	4125	5685	43	44	34	29	30	180
2	80	173	26,73	17	660	2970	1080	975	0	1560	4125	5685	35	24	35	24	16	134
2	55	163	20,70	17	853	2325	270	2253	0	2830	2871	5701	48	47	39	34	33	201
1	56	174	18,50	16	2880	990	405	1440	4320	405	990	5715	36	43	12	22	16	129
2	55	171	18,81	17	4238	577,5	210	693	0	290	5429	5719	39	40	41	32	28	180
1	78	185	22,79	17	792	3735	660	544,5	0	2910	2822	5732	24	23	28	20	19	114
2	53	170	18,34	15	1980	1584	600	1584	0	600	5148	5748	49	45	36	32	15	177
1	65	185	18,99	15	5473	93	85	133	5460	225	99	5784	29	29	24	17	16	115
1	56	177	17,87	15	4872	396	0	528	2880	1200	1716	5796	40	45	41	33	26	185
2	57	168	20,20	15	2145	693	1050	1908	2370	1050	2376	5796	49	42	42	30	18	181
2	58	169	20,31	17	1349	3474	990	0	720	4070	1023	5813	29	31	37	26	18	141
2	53	168	18,78	16	1668	2376	660	1116	720	1140	3960	5820	32	38	29	24	29	152
2	55	166	19,96	17	3480	1238	165	956	2190	2345	1304	5839	39	32	31	30	16	148
2	48	169	16,63	16	0	2670	1800	1390	870	4000	990	5860	49	41	42	35	35	202
2	66	173	22,05	17	0	4557	560	756	360	3500	2013	5873	49	49	42	35	35	210
2	51	160	19,92	15	2454	132	2980	328,5	1350	4000	544,5	5895	42	43	42	33	28	188
1	70	175	22,86	17	2908	330	1365	1293	1260	2705	1931	5896	28	28	24	20	20	120
2	56	165	20,57	16	2016	2772	420	693	630	420	4851	5901	42	40	39	35	20	176
1	67	182	20,23	17	0	2772	490	2640	2160	970	2772	5902	45	46	35	31	33	190
2	51	170	17,65	18	2843	990	440	1635	2430	920	2558	5908	35	29	29	28	21	142
2	60	176	19,37	18	853	4485	290	289,5	0	5010	907,5	5918	45	43	34	29	32	183
2	43	160	16,80	15	3976	1317	270	360	300	2950	2673	5923	43	46	35	30	24	178
2	55	176	17,76	15	898	796,5	2655	1590	1800	3265	874,5	5940	31	37	41	26	9	144

2	55	162	20,96	16	0	2973	1160	1838	1080	4660	231	5971	39	36	34	24	20	153
1	74	179	23,10	17	3120	1374	85	1410	4170	1225	594	5989	43	44	26	33	27	173
2	68	177	21,71	17	412,5	1617	205	3773	3360	205	2442	6007	41	41	42	32	26	182
2	56	162	21,34	17	0	3146	420	2448	1350	2040	2624	6014	38	30	33	24	17	142
2	45	172	15,21	17	0	4320	1575	120	0	6015	0	6015	25	24	6	15	14	84
1	56	176	18,08	15	3190	1155	270	1422	600	2830	2607	6037	40	41	27	24	22	154
1	95	177	30,32	16	4611	49,5	190	1202	4740	570	742,5	6053	35	27	13	24	23	122
2	65	165	23,88	16	2141	1476	660	1776	2070	2300	1683	6053	42	37	40	31	31	181
1	47	178	14,83	15	3433	0	840	1782	3360	880	1815	6055	40	41	41	25	25	172
1	66	176	21,31	15	3833	1965	125	133	3300	2525	231	6056	42	40	36	31	30	179
1	78	186	22,55	17	33	1386	2245	2406	840	2425	2805	6070	42	33	40	35	30	180
1	59	171	20,18	16	1733	3906	60	372	60	2760	3251	6071	34	39	30	26	28	157
1	65	187	18,59	17	5733	93	150	99	2100	3810	165	6075	29	38	22	22	17	128
1	61	170	21,11	17	3992	645	630	810	2400	2060	1617	6077	43	41	27	33	24	168
1	51	164	18,96	17	4590	924	40	528	0	3640	2442	6082	24	28	22	14	12	100
2	52	165	19,10	16	4824	693	280	297	3900	280	1914	6094	37	33	20	23	20	133
1	60	168	21,26	17	2880	0	1440	1782	0	4320	1782	6102	32	30	27	23	24	136
2	57	165	20,94	17	918	2772	1080	1332	540	1800	3762	6102	26	20	19	16	12	93
1	60	184	17,72	18	3133	0	0	2970	0	3100	3003	6103	42	40	32	31	29	174
1	55	179	17,17	16	1813	0	820	3480	3840	1580	693	6113	19	17	14	11	9	70
2	55	160	21,48	16	0	1806	1680	2646	1260	2100	2772	6132	36	29	34	28	16	143
2	47	164	17,47	17	5292	49,5	255	540	4860	1095	181,5	6137	38	38	35	26	25	162
1	66	181	20,15	17	3833	165	1980	180	3780	2180	198	6158	49	44	32	25	30	180
1	75	180	23,15	17	3160	66	160	2790	4920	200	1056	6176	34	35	31	19	23	142
1	72	178	22,72	16	898	435	705	4164	2760	2815	627	6202	44	34	23	26	18	145
2	51	166	18,51	15	1962	3150	762,5	342	930	1723	3564	6217	29	28	31	27	25	140
1	80	176	25,83	18	3450	0	2780	0	3450	2780	0	6230	28	30	25	21	20	124
1	77	181	23,50	17	810	528	180	4719	3450	180	2607	6237	45	48	42	32	18	185
1	85	185	24,84	16	3600	891	240	1568	1320	3840	1139	6299	34	33	28	23	18	136
1	87	190	24,10	17	3180	1395	90	1638	0	2970	3333	6303	44	40	37	32	25	178
1	66	182	19,93	16	3756	2079	480	0	2700	480	3135	6315	35	37	36	26	23	157
2	53	160	20,70	17	0	198	0	6120	3900	240	2178	6318	39	35	42	28	13	157
2	50	178	15,78	17	4860	249	990	223	1590	2620	2112	6322	33	40	38	34	22	167
2	52	165	19,10	17	660	3564	540	1584	0	540	5808	6348	26	18	39	26	18	127
1	62	178	19,57	17	1805	2376	1680	528	810	2180	3399	6389	29	23	20	22	26	120
1	52	165	19,10	17	3615	462	360	1962	4140	840	1419	6399	49	49	33	35	28	194
2	59	163	22,21	16	5679	742,5	0	0	5250	0	1172	6422	43	44	40	32	32	191
2	48	159	18,99	17	810	2376	540	2700	1530	540	4356	6426	32	28	16	17	25	118
1	80	180	24,69	15	0	4158	490	1782	0	490	5940	6430	32	35	30	24	27	148
2	48	165	17,63	15	0	33	6400	0	0	6400	33	6433	49	45	37	35	35	201
2	69	165	25,34	17	1310	1080	2520	1554	1140	4400	924	6464	15	27	10	12	15	79
2	60	172	20,28	17	0	522	5250	702	0	5550	924	6474	42	47	36	32	32	189
1	54	165	19,83	16	3864	1020	630	982	2520	2590	1386	6496	42	39	24	27	21	153
2	63	170	21,80	16	4755	660	190	891	0	3790	2706	6496	28	21	29	19	18	115
2	58	171	19,84	16	1709	2145	1225	1422	1440	2305	2756	6501	42	35	34	28	32	171
1	70	186	20,23	17	1400	2751	630	1726	300	4590	1617	6507	46	45	29	31	31	182
2	53	167	19,00	15	5805	0	0	720	1980	2400	2145	6525	39	28	24	23	21	135
1	67	175	21,88	17	0	2376	0	4158	0	0	6534	6534	15	15	12	12	10	64
1	70	175	22,86	17	3975	1386	630	568,5	1260	3270	2030	6560	44	42	42	35	18	181
2	65	169	22,76	16	880	346,5	30	5305	3960	110	2492	6562	35	33	41	29	32	170
2	48	158	19,23	15	4858	808,5	490	411	600	770	5198	6568	27	35	41	28	16	147
2	62	164	23,05	17	4200	924	540	937,5	4560	540	1502	6602	36	27	38	29	17	147
1	52	172	17,58	16	6045	0	560	0	3900	560	2145	6605	44	42	33	30	26	175
2	57	172	19,27	16	806	1323	900	3578	3300	1970	1337	6607	42	42	41	31	20	176
2	50	163	18,82	16	1964	1386	420	2850	2520	420	3680	6620	23	24	40	22	8	117
1	70	181	21,37	16	2295	2309	70	1954	2160	3230	1238	6628	42	46	40	29	21	178
1	52	161	20,06	16	2355	1650	310	2328	2490	1150	3003	6643	49	46	40	35	35	205
1	68	168	24,09	16	4452	396	855	952,5	240	2835	3581	6656	35	36	27	26	20	144
2	49	164	18,22	18	4158	792	120	1590	1260	120	5280	6660	30	30	24	28	6	118
2	57	160	22,27	17	4658	330	330	1350	2160	3930	577,5	6668	30	30	42	35	6	143
2	75	165	27,55	17	533	3405	910	1820	1200	5270	198	6668	34	36	22	22	18	132
2	69	185	20,16	16	1110	462	1860	3236	2640	2180	1848	6668	47	45	36	32	31	191
1	68	182	20,53	16	6318	93	125	133	1140	5265	264	6669	49	49	42	34	35	209
2	53	167	19,00	17	1980	4158	540	0	0	540	6138	6678	42	34	41	23	26	166
2	52	164	19,33	15	795	3003	180	2706	90	720	5874	6684	29	19	40	23	12	123
1	56	169	19,61	17	3690	1980	1050	0	0	1770	4950	6720	36	36	29	28	25	154
1	93	177	29,68	16	5798	693	0	247,5	1080	560	5099	6739	44	39	22	26	24	155
2	53	165	19,47	17	702	4425	935	702	120	2255	4389	6764	38	28	37	29	23	155
2	60	166	21,77	16	3492	330	1160	1806	300	2000	4488	6788	41	39	20	26	19	145

2	50	162	19,05	17	5118	231	1380	66	0	2340	4455	6795	30	20	28	16	25	119
1	68	167	24,38	16	3828	1848	1125	0	1980	1125	3696	6801	38	38	22	23	17	138
2	53	164	19,71	16	4860	693	120	1158	2010	960	3861	6831	43	40	30	30	28	171
1	67	180	20,68	15	0	4158	30	2657	0	1070	5775	6845	40	41	38	31	32	182
1	70	182	21,13	16	3657	1245	225	1719	3780	2175	891	6846	41	40	37	29	25	172
1	50	170	17,30	15	0	2475	1920	2475	0	1920	4950	6870	31	35	29	24	24	143
1	75	185	21,91	17	1980	495	240	4160	3120	1280	2475	6875	29	32	36	18	12	127
1	76	175	24,82	17	2692	462	1240	2490	2520	3440	924	6884	22	21	42	20	16	121
2	58	162	22,10	16	4380	49,5	690	1782	1440	1650	3812	6902	41	22	19	18	23	123
2	66	173	22,05	17	4370	2310	70	181,5	0	1470	5462	6932	15	14	20	14	11	74
2	65	164	24,17	16	3000	1485	210	2274	2370	210	4389	6969	41	37	38	32	17	165
1	66	172	22,31	16	6398	306	180	133	1740	1020	4257	7017	35	39	28	26	27	155
2	49	162	18,67	16	5904	198	420	495	4320	420	2277	7017	30	28	26	22	23	129
2	53	167	19,00	17	80	462	1820	4680	3120	3460	462	7042	44	48	40	35	33	200
2	60	172	20,28	17	2736	1617	2235	468	630	2235	4191	7056	28	23	29	21	16	117
2	50	172	16,90	15	2016	2193	910	1954	1080	4310	1683	7073	31	37	33	34	26	161
1	75	180	23,15	17	0	4158	1015	1902	0	2455	4620	7075	47	49	26	33	33	188
2	78	173	26,06	15	3211	3003	30	849	3360	70	3663	7093	30	27	24	20	21	122
1	58	173	19,38	16	3255	1782	630	1434	1260	2310	3531	7101	34	38	30	25	20	147
2	48	163	18,07	17	0	1584	2330	3198	3000	2330	1782	7112	30	34	35	27	14	140
2	49	167	17,57	16	2940	577,5	40	3564	780	880	5462	7122	30	31	33	20	23	137
2	53	167	19,00	16	5874	990	160	99	1680	3760	1683	7123	39	32	37	27	30	165
1	67	176	21,63	16	2430	1281	1585	1833	2250	3955	924	7129	49	49	42	35	33	208
2	55	162	20,96	15	6570	148,5	60	360	360	3660	3119	7139	32	32	32	29	12	137
1	72	185	21,04	16	3136	607,5	2020	1377	2700	3500	940,5	7141	43	47	36	33	27	186
1	62	173	20,72	15	0	1782	1620	3748	2520	1660	2970	7150	49	49	41	35	34	208
2	50	153	21,36	16	2226	1386	420	3150	2280	1140	3762	7182	45	41	17	31	5	139
2	54	166	19,60	16	1435	3423	490	1841	210	1550	5429	7189	35	35	42	32	21	165
1	58	175	18,94	16	2715	1455	585	2468	2700	2625	1898	7223	31	25	33	17	19	125
1	55	177	17,56	17	5655	1410	0	165	900	4020	2310	7230	48	41	38	30	30	187
2	43	169	15,06	15	0	0	1500	5740	3360	3880	0	7240	47	40	39	34	35	195
2	60	174	19,82	17	5593	247,5	90	1314	6120	250	874,5	7245	40	33	33	24	30	160
2	65	174	21,47	16	120	4158	0	2970	120	0	7128	7248	17	29	36	26	24	132
2	60	172	20,28	16	3534	876	840	2018	4020	1070	2178	7268	44	46	32	31	28	181
2	68	165	24,98	17	4500	924	1680	180	360	6000	924	7284	42	37	37	30	6	152
2	50	164	18,59	17	1092	4158	880	1170	1560	1120	4620	7300	33	30	34	26	20	143
1	65	175	21,22	16	0	4158	1025	2147	1080	1745	4505	7330	42	47	22	32	23	166
2	66	180	20,37	17	0	198	7140	0	0	7140	198	7338	32	33	31	24	25	145
1	71	172	24,00	16	0	1746	0	5598	5400	360	1584	7344	49	49	42	35	33	208
2	66	172	22,31	15	0	2376	1020	3948	1260	1860	4224	7344	48	44	42	35	30	199
1	96	186	27,75	16	1860	4158	760	577,5	1620	1000	4736	7356	45	44	38	27	21	175
2	54	165	19,83	17	453	4158	2005	751	420	2525	4422	7367	35	23	30	16	17	121
1	75	170	25,95	17	0	3003	1275	3097	960	1795	4620	7375	44	44	39	28	25	180
2	66	164	24,54	17	4170	1386	917,5	915	420	2118	4851	7389	39	26	20	20	7	112
1	52	183	15,53	17	0	4158	0	3240	3240	0	4158	7398	49	45	12	29	29	164
1	69	183	20,60	15	0	3375	190	3834	2880	3430	1089	7399	46	44	33	33	13	169
2	80	175	26,12	16	1215	4158	285	1782	0	1005	6435	7440	39	27	40	27	7	140
2	58	161	22,38	16	2645	462	1005	3333	1800	2345	3300	7445	25	30	41	24	16	136
2	59	169	20,66	17	5200	1458	250	576	3240	2660	1584	7484	18	15	38	24	5	100
1	63	163	23,71	15	0	3480	1105	2920	840	6665	0	7505	29	34	27	29	28	147
1	51	164	18,96	16	0	594	2035	4896	2520	2035	2970	7525	38	39	30	34	27	168
2	57	167	20,44	17	0	1842	4285	1407	0	4465	3069	7534	21	31	34	19	25	130
1	90	185	26,30	17	2880	346,5	0	4320	7200	0	346,5	7547	49	49	42	35	35	210
1	73	185	21,33	16	0	7560	0	0	0	7560	0	7560	31	31	24	23	20	129
2	56	164	20,82	18	2595	1628	3025	330	0	5515	2063	7578	47	41	42	31	31	192
1	60	178	18,94	17	0	4758	320	2502	0	1640	5940	7580	40	39	32	29	29	169
2	61	169	21,36	17	3648	165	450	3348	4320	750	2541	7611	44	43	42	33	31	193
1	57	180	17,59	17	565	1980	360	4708	3600	680	3333	7613	49	49	42	35	35	210
2	64	169	22,41	16	2310	2376	545	2382	810	1985	4818	7613	34	33	40	30	26	163
2	63	170	21,80	18	495	4158	990	1983	810	1470	5346	7626	49	49	42	35	24	199
2	42	150	18,67	15	2583	1658	1980	1410	2970	3060	1601	7631	49	49	32	30	35	195
2	70	176	22,60	17	5130	0	840	1680	3840	840	2970	7650	32	38	41	32	14	157
1	87	180	26,85	18	3313	93	3040	1209	3300	3860	495	7655	24	24	24	23	23	118
1	61	178	19,25	16	2645	3486	1400	132	300	3700	3663	7663	30	34	42	31	6	143
2	74	174	24,44	17	2946	693	390	3638	4560	2150	957	7667	49	49	35	32	30	195
1	63	179	19,66	16	6315	990	180	198	1800	3540	2343	7683	39	44	28	34	24	169
1	57	170	19,72	16	2185	4680	480	372	0	5440	2277	7717	48	49	41	33	32	203
1	53	175	17,31	17	0	1386	1350	4992	3240	2310	2178	7728	33	25	9	15	20	102
2	56	172	18,93	17	6588	0	450	742,5	5400	450	1931	7781	44	42	35	31	22	174

1	55	160	21,48	15	3600	1188	0	3000	1560	5040	1188	7788	49	49	42	35	29	204
2	49	164	18,22	17	179	249	705	6665	3780	2335	1683	7798	24	24	22	19	19	108
2	51	153	21,79	16	6500	495	840	0	420	5880	1535	7835	47	43	20	28	10	148
2	53	175	17,31	16	2080	4158	40	1578	0	2840	5016	7856	26	31	30	20	14	121
1	75	178	23,67	16	6213	462	345	873	2340	3705	1848	7893	43	38	30	28	23	162
2	95	177	30,32	17	0	4158	3780	0	0	3780	4158	7938	25	22	20	22	8	97
2	50	169	17,51	17	7448	396	120	0	2940	3720	1304	7964	10	9	37	10	5	71
1	75	187	21,45	16	100	2358	360	5196	3300	4120	594	8014	39	39	34	31	14	157
1	83	173	27,73	15	3600	396	3300	720	4320	3300	396	8016	38	40	37	28	25	168
1	78	183	23,29	17	4263	2574	420	780	1200	1260	5577	8037	34	36	24	22	17	133
2	81	181	24,72	17	4707	2628	330	396	810	5370	1881	8061	21	22	34	16	13	106
2	54	164	20,08	17	7765	165	0	132	7560	40	462	8062	42	39	35	27	27	170
2	60	167	21,51	17	6570	742,5	560	198	0	4160	3911	8071	28	37	40	34	13	152
2	54	162	20,58	18	3990	2430	900	756	1800	3900	2376	8076	20	32	28	31	11	122
1	68	179	21,22	18	4320	165	0	3600	7200	720	165	8085	48	46	21	32	24	171
2	60	170	20,76	15	1187	3633	1960	1344	1080	5740	1304	8124	33	38	39	24	13	147
2	55	166	19,96	17	4582	1155	1260	1155	0	4060	4092	8152	25	31	33	21	25	135
1	58	180	17,90	17	2160	3003	0	3003	2160	0	6006	8166	40	34	20	19	23	136
2	63	169	22,06	18	2062	3564	930	1617	0	1210	6963	8173	29	30	40	24	16	139
2	65	165	23,88	17	4170	1386	1055	1575	420	2255	5511	8186	28	30	38	21	20	137
2	50	171	17,10	16	3338	2145	900	1822	3180	1180	3845	8205	45	42	16	31	20	154
2	50	155	20,81	17	4180	2393	280	1361	4320	560	3333	8213	47	39	17	26	24	153
2	42	165	15,43	15	4878	792	2070	476	0	2870	5346	8216	32	24	38	25	10	129
2	60	180	18,52	16	3006	2079	270	2862	510	1470	6237	8217	40	30	33	23	26	152
2	53	165	19,47	17	1782	3003	560	2877	1260	560	6402	8222	49	49	42	33	32	205
2	49	164	18,22	17	5904	396	1740	198	2160	3900	2178	8238	30	34	38	23	26	151
1	63	183	18,81	15	1911	4722	1340	297	1440	4520	2310	8270	40	43	27	32	26	168
2	54	160	21,09	16	4140	1155	1760	1215	600	5360	2310	8270	41	31	37	29	19	157
2	44	156	18,08	17	5993	1238	1020	33	0	4620	3663	8283	37	45	34	29	19	164
2	46	155	19,15	16	4283	2622	1400	0	0	5120	3185	8305	30	34	32	28	24	148
2	87	168	30,82	17	6242	462	490	1113	4320	1050	2937	8307	38	42	38	32	15	165
2	49	160	19,14	16	1040	4158	1800	1312	0	2320	5990	8310	34	34	37	22	23	150
1	72	182	21,74	18	4718	837	630	2160	1080	2810	4455	8345	38	43	12	22	24	139
2	60	170	20,76	16	5718	2391	90	148,5	1440	2370	4538	8348	34	37	34	26	20	151
1	84	180	25,93	17	4165	1386	270	2556	2460	670	5247	8377	40	36	42	35	13	166
1	70	175	22,86	17	0	2772	2730	2880	2880	2730	2772	8382	43	45	18	33	31	170
2	50	166	18,14	17	4671	1320	0	2424	4140	1800	2475	8415	34	31	12	26	10	113
2	68	169	23,81	17	660	2079	420	5256	2700	1260	4455	8415	44	41	42	32	13	172
2	50	157	20,28	18	3150	4158	540	577,5	1440	1260	5726	8426	35	36	29	28	16	144
1	66	180	20,37	16	2805	1733	0	3893	3150	660	4620	8430	49	45	42	35	30	201
1	62	187	17,73	15	2304	2268	1080	2826	0	4320	4158	8478	33	38	12	21	23	127
1	73	175	23,84	16	0	1287	5940	1260	1260	5940	1287	8487	40	47	31	29	24	171
2	60	168	21,26	17	0	4158	180	4158	0	180	8316	8496	8	12	35	11	13	79
1	55	165	20,20	15	5410	462	730	1897	2160	1290	5049	8499	29	34	10	17	21	111
2	55	171	18,81	17	6318	858	1005	318	2280	1005	5214	8499	31	47	27	34	20	159
2	51	173	17,04	17	937,5	4158	960	2466	1080	1320	6122	8522	30	29	38	21	11	129
2	58	165	21,30	17	5618	0	1430	1485	1740	2470	4323	8533	38	37	38	27	20	160
1	60	161	23,15	17	0	3315	2250	2970	2160	4890	1485	8535	38	42	33	25	25	163
2	63	169	22,06	16	7980	495	60	0	3600	2460	2475	8535	34	36	31	28	27	156
1	60	177	19,15	16	1314	4158	950	2129	1080	1910	5561	8551	40	36	31	30	28	165
2	54	164	20,08	17	6540	1302	70	693	5460	1330	1815	8605	44	40	20	26	24	154
2	59	164	21,94	16	4433	1328	420	2436	3990	1640	2987	8617	39	37	37	33	27	173
2	52	160	20,31	16	5260	1155	1020	1188	2250	1060	5313	8623	49	49	42	35	29	204
2	55	173	18,38	16	0	4158	420	4052	1200	500	6930	8630	48	49	41	35	24	197
1	77	182	23,25	18	4178	1250	570	2640	4320	2700	1617	8637	44	40	29	27	18	158
2	60	162	22,86	16	6810	945	200	720	960	4250	3465	8675	35	33	28	26	24	146
2	53	158	21,23	18	5130	3564	0	0	2160	0	6534	8694	14	18	36	13	14	95
2	60	173	20,05	17	5835	247,5	100	2528	5400	1660	1650	8710	49	49	39	33	29	199
1	80	176	25,83	17	0	3537	962,5	4230	2070	6363	297	8730	24	21	23	22	16	106
1	45	167	16,14	16	0	4158	1548	3045	900	1548	6303	8751	35	23	16	17	28	119
2	56	171	19,15	17	3072	3456	0	2226	840	2040	5874	8754	36	43	39	32	13	163
2	67	173	22,39	17	1190	495	3720	3382	1080	4440	3267	8787	49	49	42	35	35	210
1	48	172	16,22	15	5577	495	320	2400	3480	3200	2112	8792	46	49	42	35	24	196
1	70	190	19,39	17	4327	924	2850	699	1350	4810	2640	8800	37	41	31	30	20	159
1	50	166	18,14	15	280	5400	505	2620	2340	6465	0	8805	29	30	29	23	20	131
2	50	163	18,82	17	3000	3444	370	1994	2880	4410	1518	8808	46	49	42	35	31	203
2	40	150	17,78	17	4858	3119	620	231	420	900	7508	8828	43	45	26	22	24	160
2	58	172	19,61	15	1155	1838	670	5198	840	2130	5891	8861	43	45	41	35	26	190
1	75	198	19,13	15	5490	2079	255	1040	1080	4385	3399	8864	36	33	26	22	28	145

1	80	180	24,69	17	5400	1617	0	1847	5400	560	2904	8864	49	49	42	32	19	191
1	90	183	26,87	16	0	2886	2475	3510	1080	6075	1716	8871	40	23	29	17	34	143
1	59	176	19,05	15	49,5	4158	1080	3594	2880	1200	4802	8882	41	44	26	27	23	161
1	84	178	26,51	17	1893	1794	180	5028	1230	1890	5775	8895	28	31	40	30	14	143
2	60	164	22,31	17	4335	1782	2240	548,5	2340	3480	3086	8906	36	38	41	32	12	159
1	55	183	16,42	15	7518	396	1000	0	3360	1000	4554	8914	46	46	39	32	28	191
2	56	170	19,38	17	3570	4158	0	1188	600	0	8316	8916	45	35	41	35	24	180
1	85	190	23,55	16	4198	1898	1050	1782	0	3350	5577	8927	32	35	30	26	24	147
2	56	165	20,57	16	0	2772	3045	3132	360	3045	5544	8949	22	32	31	24	19	128
2	58	183	17,32	16	2988	3330	1760	876	120	7200	1634	8954	28	29	9	26	19	111
1	83	184	24,52	17	3885	600	90	4380	7500	1290	165	8955	49	49	23	31	29	181
2	66	161	25,46	17	6477	1617	720	175,5	1320	4320	3350	8990	48	45	40	35	27	195
2	48	163	18,07	16	5410	247,5	390	2952	1020	1990	5990	9000	41	38	35	31	27	172
1	69	175	22,53	17	4050	3150	360	1440	1080	3960	3960	9000	45	47	15	28	24	159
2	56	172	18,93	16	3871	1674	0	3466	3900	2240	2871	9011	43	43	29	27	12	154
2	60	175	19,59	17	3450	3015	700	1879	3060	5060	924	9044	43	43	39	33	25	183
2	56	170	19,38	16	0	1188	1080	6798	2160	1560	5346	9066	41	43	38	32	21	175
1	60	178	18,94	17	3465	2541	1100	1970	660	1420	6996	9076	35	44	23	21	15	138
2	44	161	16,97	17	6548	1485	70	994	0	4790	4307	9097	24	25	40	23	16	128
2	68	174	22,46	17	2880	1188	5040	0	0	7920	1188	9108	25	26	24	24	15	114
2	63	173	21,05	15	4335	99	450	4236	3510	2970	2640	9120	37	39	38	27	18	159
2	65	171	22,23	16	8192	378	350	202,5	7560	1150	412,5	9123	37	42	30	32	24	165
2	59	170	20,42	17	720	2106	360	5937	5040	1080	3003	9123	45	47	41	33	35	201
1	93	198	23,72	17	3693	4746	240	445,5	0	5560	3564	9124	42	34	13	14	27	130
2	60	170	20,76	17	2425	4158	180	2376	720	400	8019	9139	38	35	33	22	19	147
1	85	188	24,05	17	678	2898	857,5	4756	4080	4318	792	9190	42	44	36	30	21	173
2	54	160	21,09	18	2980	1413	245	4557	4380	1845	2970	9195	24	30	27	19	20	120
2	53	168	18,78	17	2880	2772	480	3096	720	3360	5148	9228	45	42	38	29	28	182
2	52	166	18,87	17	2970	3564	870	1840	0	2710	6534	9244	45	44	32	25	27	173
2	54	156	22,19	16	6588	66	1570	1027	3930	2730	2591	9251	28	28	24	20	20	120
2	58	168	20,55	17	4968	774	960	2565	3780	2550	2937	9267	42	36	38	31	26	173
1	64	172	21,63	17	4455	2772	990	1116	1800	3870	3663	9333	29	27	40	20	18	134
2	60	163	22,58	16	7038	1155	1140	0	0	4020	5313	9333	41	41	34	27	27	170
2	48	155	19,98	17	0	2079	0	7283	3240	0	6122	9362	40	36	34	26	25	161
2	40	160	15,63	17	2772	3168	960	2475	0	960	8415	9375	22	17	17	22	17	95
1	55	175	17,96	16	4275	1566	0	3546	2160	3300	3927	9387	49	49	42	35	35	210
2	62	171	21,20	16	0	8369	280	742,5	0	7840	1551	9391	26	18	31	20	14	109
2	56	167	20,08	16	4075	990	2010	2320	4590	2330	2475	9395	44	46	37	31	26	184
2	50	165	18,37	16	5958	693	0	2772	1800	0	7623	9423	43	45	39	28	12	167
2	63	182	19,02	15	2080	2247	1260	3874	2160	5090	2211	9461	34	27	11	20	9	101
2	50	156	20,55	17	66	693	240	8478	0	4560	4917	9477	22	18	36	20	12	108
1	67	178	21,15	15	4425	2853	630	1582	0	7510	1980	9490	48	43	42	33	9	175
2	63	163	23,71	15	3862	3369	925	1351	840	6225	2442	9507	27	28	37	23	23	138
1	64	169	22,41	17	330	1271	210	7700	5040	890	3581	9511	49	49	42	35	26	201
2	56	172	18,93	16	5778	2376	0	1360	0	1660	7854	9514	35	36	35	27	22	155
1	65	183	19,41	17	2520	4158	40	2808	4140	40	5346	9526	46	46	36	23	16	167
2	54	165	19,83	17	3867	2772	270	2622	4500	510	4521	9531	36	41	19	15	24	135
2	68	165	24,98	16	8370	165	325	720	6120	325	3135	9580	25	28	27	23	14	117
2	70	170	24,22	16	3776	4353	1410	66	1440	7010	1155	9605	32	25	42	27	25	151
2	55	167	19,72	16	429	2970	5400	813	780	5400	3432	9612	35	20	29	21	18	123
2	65	169	22,76	15	1965	1733	2640	3278	3600	3540	2475	9615	35	20	41	19	5	120
2	59	170	20,42	17	8172	198	540	720	4920	1740	2970	9630	38	44	38	25	22	167
2	53	161	20,45	17	2955	3003	0	3675	4320	0	5313	9633	37	32	40	31	22	162
2	78	165	28,65	17	4675	1584	1610	1769	4680	2070	2888	9638	35	34	39	26	21	155
2	59	171	20,18	17	462	924	0	8253	7560	0	2079	9639	38	40	31	30	21	160
1	68	183	20,31	16	1271	3171	0	5200	3120	5020	1502	9642	38	42	26	29	20	155
2	50	165	18,37	16	4530	2772	360	2034	2520	840	6336	9696	42	40	33	30	22	167
1	95	186	27,46	16	9105	429	120	99	3360	3720	2673	9753	13	21	28	12	18	92
1	115	198	29,33	17	2520	4158	270	2808	4140	270	5346	9756	47	39	39	23	26	174
1	74	177	23,62	17	6117	346,5	512,5	2799	5400	2313	2063	9775	49	43	39	34	20	185
2	59	172	19,94	16	1130	693	60	7893	4320	3740	1716	9776	42	47	42	30	22	183
1	65	175	21,22	17	4884	4248	300	410	420	1370	8052	9842	35	26	34	24	18	137
2	46	166	16,69	17	2511	4158	660	2526	2520	900	6435	9855	12	17	26	10	6	71
1	53	170	18,34	15	7930	495	975	462	4320	1615	3927	9862	42	38	33	30	30	173
2	65	173	21,72	18	6438	2079	980	372	840	2660	6369	9869	45	39	36	27	19	166
1	63	185	18,41	16	3811	3213	1225	1631	2700	6025	1155	9880	46	37	27	26	23	159
2	48	157	19,47	16	4638	2772	490	1980	720	1570	7590	9880	46	44	35	32	28	185
2	58	168	20,55	17	3975	247,5	945	4716	6030	1725	2129	9884	49	49	30	35	31	194
1	64	184	18,90	17	5400	1134	0	3356	4320	4860	709,5	9890	43	46	39	32	28	188

2	66	171	22,57	16	2828	3003	1075	3003	2160	1495	6254	9909	38	35	36	34	22	165
2	59	170	20,42	18	3375	693	660	5184	2700	2460	4752	9912	43	38	41	22	12	156
2	67	164	24,91	18	5866	2772	180	1134	5940	580	3432	9952	39	30	31	17	24	141
1	68	179	21,22	16	0	3003	1235	5718	1080	1715	7161	9956	38	32	39	30	25	164
1	54	179	16,85	15	3540	3003	2415	1020	1830	4815	3333	9978	43	35	30	26	13	147
2	54	171	18,47	16	4746	2003	635	2610	5160	2375	2459	9994	48	46	39	35	31	199
1	68	182	20,53	17	3274	787,5	1500	4434	4560	4000	1436	9996	16	29	26	14	9	94
1	61	170	21,11	18	8645	519	140	702	3900	3400	2706	10006	49	48	40	34	29	200
2	70	160	27,34	17	5598	1188	3250	0	0	4690	5346	10036	29	31	38	29	20	147
1	84	187	24,02	18	2160	2475	4260	1188	2160	4260	3663	10083	38	46	29	29	30	172
1	100	191	27,41	17	3450	4932	270	1440	1440	2910	5742	10092	49	36	42	33	32	192
2	61	169	21,36	17	2826	1071	750	5466	5040	2070	3003	10113	39	41	34	24	25	163
1	72	170	24,91	15	513	7398	1800	411	60	5640	4422	10122	27	28	23	16	20	114
2	68	170	23,53	16	0	3465	2520	4158	0	2520	7623	10143	49	39	33	33	30	184
1	74	175	24,16	18	8303	0	0	1887	4170	1400	4620	10190	47	49	39	32	27	194
2	44	162	16,77	15	0	1155	4420	4678	0	4940	5313	10253	33	33	31	26	23	146
1	73	175	23,84	17	427,5	4148	610	5082	2100	7210	957	10267	47	46	40	35	30	198
1	68	182	20,53	18	3388	2358	805	3720	4440	4445	1386	10271	42	45	39	35	26	187
1	67	185	19,58	16	3010	6093	70	1133	660	5950	3696	10306	42	37	41	26	16	162
2	63	164	23,42	17	6240	2871	1200	0	1980	4800	3531	10311	37	38	41	31	23	170
2	54	161	20,83	16	2414	5046	720	2137	1350	2350	6617	10317	40	47	27	30	25	169
2	53	176	17,11	16	3055	4158	120	3003	630	400	9306	10336	25	17	29	22	12	105
1	64	179	19,97	17	4320	4428	895	700	0	5525	4818	10343	36	29	31	27	20	143
1	62	178	19,57	17	3250	4017	3130	0	0	7130	3267	10397	36	30	27	19	13	125
2	75	178	23,67	16	2376	594	55	7380	5400	55	4950	10405	41	49	29	32	28	179
1	70	175	22,86	17	5670	825	800	3120	5820	800	3795	10415	46	44	38	30	26	184
2	63	166	22,86	15	7434	297	1230	1470	5010	1230	4191	10431	26	26	32	24	17	125
2	62	172	20,96	17	3880	6603	0	0	3600	3880	3003	10483	45	45	25	29	29	173
1	65	181	19,84	17	3735	3443	150	3240	6480	2850	1238	10568	48	43	30	34	29	184
1	73	171	24,96	16	5073	3186	2030	300	2700	7130	759	10589	26	35	19	24	10	114
2	50	160	19,53	17	0	5238	1110	4314	360	2910	7392	10662	43	39	33	30	21	166
2	57	162	21,72	17	4536	4158	1980	0	0	4140	6534	10674	30	38	40	29	23	160
1	76	185	22,21	17	5772	2772	0	2160	4320	840	5544	10704	49	49	42	35	35	210
2	74	166	26,85	17	1493	7278	442,5	1506	2520	6533	1667	10719	44	37	38	31	28	178
2	59	172	19,94	16	8430	1782	0	528	5460	0	5280	10740	36	44	42	35	27	184
2	68	170	23,53	16	1335	2502	1830	5073	5040	3390	2310	10740	48	48	33	27	26	182
2	50	161	19,29	17	5958	1980	660	2152	1980	1840	6930	10750	39	30	37	28	21	155
2	54	163	20,32	16	4172	2772	480	3348	3240	800	6732	10772	49	39	42	30	35	195
1	60	169	21,01	17	5306	693	0	4782	7200	1040	2541	10781	38	40	36	22	24	160
1	88	202	21,57	17	5853	924	1980	2040	5400	3780	1617	10797	30	30	27	16	19	122
1	55	179	17,17	16	8000	573	1315	922,5	5580	4455	775,5	10811	44	48	32	29	25	178
2	87	169	30,46	16	6092	3234	375	1118	4440	1775	4604	10819	8	10	32	16	7	73
2	46	165	16,90	17	6465	412,5	1018	2938	3900	4458	2475	10833	31	36	21	28	19	135
1	80	178	25,25	17	2434	594	887,5	6924	7650	1408	1782	10840	45	39	41	26	27	178
1	60	190	16,62	17	0	3492	4640	2720	720	6320	3812	10852	47	45	22	29	30	173
2	41	161	15,82	15	0	5067	4395	1475	0	8495	2442	10937	32	40	27	33	23	155
2	55	163	20,70	16	5072	2426	220	3226	2580	1780	6584	10944	47	47	40	34	26	194
1	75	178	23,67	16	520	4158	0	6318	0	2680	8316	10996	37	40	33	27	24	161
1	64	178	20,20	16	7044	2574	990	396	1080	3390	6534	11004	35	40	33	31	25	164
2	48	156	19,72	16	8010	1386	1440	198	4320	4800	1914	11034	37	31	34	27	22	151
1	70	181	21,37	16	5250	0	1350	4455	7230	1350	2475	11055	43	44	36	31	31	185
1	51	172	17,24	16	8730	1494	225	646,5	5700	1485	3911	11096	40	45	16	17	18	136
2	58	176	18,72	15	3960	2457	1833	2877	5220	3993	1914	11127	49	49	42	35	35	210
1	65	177	20,75	17	9639	462	360	702	6480	1680	3003	11163	39	36	35	23	26	159
2	56	158	22,43	17	10080	33	1080	0	6480	4680	33	11193	18	22	28	20	7	95
2	75	162	28,58	16	2390	6258	850	1710	720	4350	6138	11208	41	36	42	31	22	172
1	65	181	19,84	17	7590	1053	1140	1428	5400	2940	2871	11211	40	44	29	28	27	168
1	59	181	18,01	15	5022	1275	1170	3840	6840	1530	2937	11307	45	42	35	31	25	178
2	70	180	21,60	15	8124	1743	450	1084	2010	4870	4521	11401	42	40	39	32	26	179
2	46	160	17,97	16	6863	2772	120	1698	2520	2960	5973	11453	33	34	17	12	7	103
2	53	155	22,06	15	0	5163	6250	133	60	8450	3036	11546	38	41	40	29	20	168
2	47	161	18,13	17	5130	4518	1140	774	1620	2220	7722	11562	27	26	19	18	19	109
2	60	169	21,01	17	9037	1617	430	501	5730	2390	3465	11585	42	42	34	29	26	173
2	50	160	19,53	17	5838	1617	0	4155	2880	1800	6930	11610	49	47	42	35	26	199
2	67	162	25,53	17	3513	2376	3535	2202	2340	5095	4191	11626	38	39	42	25	26	170
1	60	177	19,15	16	7097	1143	1500	1928	6120	4310	1238	11668	45	39	23	30	29	166
1	82	184	24,22	16	8244	2970	110	429	1080	3710	6963	11753	24	20	14	23	13	94
1	69	165	25,34	17	4320	0	2190	5256	4320	5070	2376	11766	32	31	34	25	25	147
2	49	166	17,78	16	3480	1782	4790	1716	1080	7190	3498	11768	14	20	35	25	14	108

2	60	168	21,26	15	5460	4158	1498	654	5520	1498	4752	11770	42	44	37	30	29	182
1	57	178	17,99	16	4119	2136	2750	2778	540	7250	3993	11783	35	30	36	34	15	150
2	50	159	19,78	16	4284	4275	1715	1512	2880	3725	5181	11786	49	49	42	35	35	210
1	99	186	28,62	16	7182	93	40	4503	5310	4660	1848	11818	43	49	42	35	31	200
2	69	178	21,78	17	0	2310	1690	7820	5250	2610	3960	11820	49	48	42	35	23	197
1	51	176	16,46	17	5162	1980	1080	3624	5940	1220	4686	11846	43	39	35	21	30	168
2	48	157	19,47	15	8018	924	1770	1191	2880	3710	5313	11903	30	33	32	23	20	138
1	80	187	22,88	17	6422	918	4500	133	3300	6000	2673	11973	47	47	36	35	34	199
1	74	186	21,39	16	7671	0	3600	720	7200	4560	231	11991	49	49	42	35	35	210
1	70	174	23,12	17	5130	3255	490	3120	3120	4750	4125	11995	47	47	37	27	27	185
2	43	159	17,01	16	6069	577,5	1570	3785	7230	1570	3201	12001	18	11	40	16	10	95
2	60	177	19,15	16	6558	1053	1155	3240	4680	2475	4851	12006	44	44	21	30	20	159
1	72	184	21,27	17	4965	2079	3630	1413	1800	6030	4257	12087	39	48	32	26	19	164
2	54	166	19,60	17	3918	3984	690	3564	3240	1590	7326	12156	39	40	31	29	29	168
1	76	185	22,21	15	4245	2253	3750	1920	4560	6750	858	12168	36	39	38	31	28	172
1	75	175	24,49	16	2930	4473	710	4067	2940	3960	5280	12180	32	27	36	31	15	141
2	55	173	18,38	16	7545	2931	780	933	6300	2820	3069	12189	21	27	24	15	16	103
2	55	168	19,49	16	6279	3114	2205	594	2520	6405	3267	12192	39	32	41	33	27	172
1	60	170	20,76	16	6051	2700	1360	2100	4200	7120	891	12211	43	49	39	35	34	200
2	35	165	12,86	15	2582	8148	1200	297	0	8300	3927	12227	39	33	39	35	26	172
1	63	171	21,55	16	10833	346,5	480	577,5	6480	4800	957	12237	32	23	42	35	5	137
1	67	173	22,39	17	5556	198	1860	4632	5040	5820	1386	12246	39	48	26	23	16	152
2	72	180	22,22	16	2473	3696	3445	2667	3450	3485	5346	12281	41	42	42	32	26	183
1	69	180	21,30	17	10332	396	180	1386	6480	1260	4554	12294	34	34	25	15	30	138
2	57	167	20,44	17	6318	3003	1000	1977	3840	1000	7458	12298	41	37	42	25	32	177
2	60	175	19,59	16	5220	1188	150	5745	4860	2790	4653	12303	43	47	41	27	28	186
1	75	170	25,95	15	3956	6762	240	1386	1680	7100	3564	12344	21	31	41	35	16	144
1	65	178	20,52	17	8030	231	660	3431	8280	2620	1452	12352	46	41	42	35	21	185
1	75	184	22,15	16	1376	4494	1350	5178	1800	2760	7838	12398	30	36	41	26	27	160
1	70	172	23,66	16	7683	1782	1800	1138	1080	5680	5643	12403	38	39	24	24	24	149
1	65	165	23,88	16	4257	4317	2355	1475	2220	6455	3729	12404	38	44	34	26	21	163
1	78	175	25,47	17	6978	148,5	3180	2115	2820	4140	5462	12422	38	39	33	27	23	160
2	45	163	16,94	17	3975	4158	800	3492	2760	4880	4785	12425	36	41	20	28	23	148
1	62	172	20,96	15	4533	453	5590	1853	1440	10890	99	12429	31	34	29	23	21	138
2	59	169	20,66	16	5895	2175	1343	3129	3000	7743	1799	12541	38	33	39	23	25	158
1	55	165	20,20	17	0	4536	1850	6162	3240	5150	4158	12548	44	48	26	32	24	174
1	84	188	23,77	18	0	9045	480	3069	1980	8040	2574	12594	40	43	33	32	26	174
1	75	180	23,15	17	7395	33	2505	2678	2910	6665	3036	12611	39	40	33	29	14	155
2	60	170	20,76	17	1910	4158	1450	5106	1080	5010	6534	12624	45	30	40	34	18	167
2	50	164	18,59	17	3765	3297	3960	1620	5040	5820	1782	12642	37	47	23	27	20	154
2	69	170	23,88	15	5103	2145	690	4734	1890	4050	6732	12672	37	40	38	30	10	155
2	55	164	20,45	16	5040	1848	1575	4212	3000	3075	6600	12675	38	36	37	24	24	159
2	50	163	18,82	15	3040	66	3105	6510	6720	3625	2376	12721	31	34	38	27	17	147
2	58	170	20,07	17	4268	4158	1960	2346	1500	4120	7112	12732	29	28	32	15	27	131
1	75	175	24,49	18	7398	3294	0	2100	5340	2700	4752	12792	27	21	32	14	20	114
1	67	180	20,68	18	4062	4158	3105	1497	840	5745	6237	12822	46	40	30	29	30	175
1	74	173	24,73	17	3386	6318	2440	693	3240	4680	4917	12837	13	12	14	17	11	67
1	69	177	22,02	17	3600	0	7690	1560	720	12130	0	12850	42	45	37	30	29	183
2	63	170	21,80	17	5988	1584	1620	3672	5760	3540	3564	12864	40	40	40	29	25	174
1	60	180	18,52	17	4044	3546	30	5280	5400	2550	4950	12900	31	38	36	28	27	160
2	47	168	16,65	17	6736	1329	0	4843	1440	5660	5808	12908	19	18	23	16	9	85
2	86	174	28,41	15	2195	2772	2865	5097	3480	3905	5544	12929	47	38	39	29	29	182
1	47	162	17,91	15	0	1350	2970	8640	6480	6480	0	12960	29	30	28	22	20	129
2	50	168	17,72	16	3983	0	3608	5400	6480	5768	742,5	12990	46	47	39	28	23	183
2	60	158	24,03	17	9924	384	1560	1182	6120	5280	1650	13050	28	24	42	21	13	128
1	76	182	22,94	17	9705	3003	210	133	5460	2410	5181	13051	39	37	30	33	23	162
2	65	168	23,03	17	5688	2888	0	4610	1380	3720	8085	13185	34	31	24	24	21	134
1	88	184	25,99	16	10170	2100	0	970	5010	5260	2970	13240	39	36	36	31	18	160
2	60	174	19,82	17	2619	2970	2210	5462	4470	3610	5181	13261	47	39	41	31	31	189
2	58	169	20,31	17	0	2970	5110	5202	3900	5950	3432	13282	47	41	35	26	23	172
1	65	173	21,72	16	3925	3633	1570	4187	5700	5750	1865	13315	47	43	42	26	18	176
2	60	169	21,01	17	1455	8946	900	2028	120	10140	3069	13329	35	34	38	25	28	160
2	56	172	18,93	16	4800	2268	3760	2592	1710	6760	4950	13420	17	22	31	14	11	95
2	55	165	20,20	16	7218	4158	750	1314	2700	1830	8910	13440	49	47	40	32	34	202
1	75	185	21,91	16	3075	3657	3035	3687	630	9755	3069	13454	49	49	41	34	34	207
1	55	176	17,76	15	0	3348	1220	8998	4320	3900	5346	13566	35	43	21	31	7	137
2	49	152	21,21	16	10278	1188	300	1818	3870	3180	6534	13584	28	33	42	27	10	140
1	97	190	26,87	18	7422	924	4080	1182	3780	6660	3168	13608	37	34	42	29	20	162
1	60	183	17,92	17	7630	4767	490	751	5670	6120	1848	13638	34	37	31	26	29	157

2	69	164	25,65	16	4825	3483	4800	585,5	1080	5700	6914	13694	11	21	32	19	17	100
2	65	166	23,59	15	6758	4158	2790	0	0	5390	8316	13706	24	29	39	32	18	142
1	69	188	19,52	15	5424	2127	3410	2748	3060	7250	3399	13709	35	37	23	21	19	135
1	75	173	25,06	16	10278	1854	1013	586,5	1320	7313	5099	13731	49	49	42	35	35	210
2	61	164	22,68	15	7826	1617	180	4182	7560	2780	3465	13805	43	43	36	30	24	176
2	54	167	19,36	18	6225	99	2875	4616	5520	4335	3960	13815	31	28	36	29	18	142
2	50	164	18,59	17	8438	2022	780	2620	6660	5500	1700	13860	42	47	42	30	23	184
2	49	161	18,90	18	0	9342	450	4083	1080	8010	4785	13875	20	17	36	20	13	106
2	69	163	25,97	17	6570	1485	4500	1328	1080	8100	4703	13883	18	12	34	26	15	105
1	60	173	20,05	17	10692	396	360	2440	8640	2080	3168	13888	35	45	21	20	21	142
2	69	176	22,28	15	6803	2772	120	4236	2640	5120	6171	13931	34	34	35	27	30	160
1	67	176	21,63	16	8496	1584	360	3492	7200	1980	4752	13932	38	40	42	25	12	157
1	60	175	19,59	16	240	5337	700	7659	7560	5980	396	13936	41	42	29	20	25	157
1	65	169	22,76	16	4218	5508	30	4218	120	1380	12474	13974	22	32	27	17	15	113
2	64	171	21,89	17	5784	2373	1250	4578	6480	4370	3135	13985	47	42	40	33	21	183
1	67	170	23,18	16	7698	2772	2135	1386	2700	2975	8316	13991	47	48	34	31	27	187
1	69	181	21,06	17	2970	3465	0	7560	7560	0	6435	13995	37	40	36	35	5	153
2	62	163	23,34	16	6398	3465	0	4158	0	2240	11781	14021	18	27	24	21	24	114
2	58	160	22,66	16	7250	1071	490	5218	4020	2650	7359	14029	43	48	36	35	27	189
1	57	168	20,20	16	8002	2145	1980	1946	3900	6180	3993	14073	40	33	37	24	26	160
2	53	167	19,00	16	8736	4158	0	1188	0	5040	9042	14082	37	35	37	33	24	166
2	45	165	16,53	16	10938	594	1485	1080	4980	4365	4752	14097	41	44	37	34	25	181
1	74	188	20,94	17	4068	6786	1863	1485	60	8103	6039	14202	23	27	40	33	17	140
1	75	184	22,15	15	9372	2772	1600	495	5400	2800	6039	14239	29	26	38	20	17	130
2	50	167	17,93	17	6408	3465	2220	2156	810	4380	9059	14249	7	8	21	14	8	58
1	76	177	24,26	17	0	4158	700	9398	4320	1620	8316	14256	40	36	26	24	18	144
1	81	183	24,19	17	3908	2517	3560	4305	4320	5020	4950	14290	34	35	37	29	11	146
1	69	172	23,32	17	6933	165	0	7236	11880	1480	973,5	14334	47	47	39	34	17	184
2	51	168	18,07	17	8175	33	280	5859	6750	5320	2277	14347	44	38	27	26	22	157
1	72	182	21,74	17	8410	4788	0	1233	6600	670	7161	14431	49	46	42	35	32	204
1	57	164	21,19	16	3465	2970	4175	3824	4620	5095	4719	14434	39	41	38	28	24	170
1	68	176	21,95	15	5229	495	4410	4305	3990	4410	6039	14439	49	49	42	35	32	207
2	45	152	19,48	15	4505	1110	5060	3764	6420	6270	1749	14439	47	48	40	34	26	195
1	55	175	17,96	15	11840	2250	60	300	7230	4910	2310	14450	47	45	42	35	34	203
1	74	180	22,84	17	12633	1845	0	0	7560	5400	1518	14478	25	26	17	14	6	88
1	65	180	20,06	16	9170	396	675	4320	5760	5435	3366	14561	32	33	32	26	23	146
2	55	166	19,96	16	5745	4245	630	3960	3960	6330	4290	14580	31	30	40	20	10	131
1	68	182	20,53	16	8083	4815	1380	325	5040	5900	3663	14603	31	37	42	31	25	166
1	64	173	21,38	18	0	5055	1400	8175	4680	8300	1650	14630	49	49	39	32	27	196
2	65	172	21,97	17	10065	33	220	4320	8640	3820	2178	14638	41	44	41	24	27	177
1	62	170	21,45	16	6360	2628	0	5662	5760	7240	1650	14650	28	37	29	26	18	138
1	66	185	19,28	16	5958	6558	520	1617	0	4720	9933	14653	42	47	23	26	20	158
2	45	152	19,48	16	10284	2004	40	2415	4560	5200	4983	14743	39	39	36	25	13	152
1	73	179	22,78	17	3791	4158	3850	3003	1080	5290	8432	14802	49	49	42	35	35	210
1	70	172	23,66	18	1902	3371	3373	6170	2160	6633	6023	14815	32	26	28	22	19	127
2	50	164	18,59	16	4758	4158	2565	3374	420	4205	10230	14855	49	49	27	32	27	184
2	60	174	19,82	16	5845	2657	225	6180	8550	1225	5132	14907	47	48	26	32	30	183
2	54	168	19,13	15	3250	9342	1190	1130	1080	9030	4802	14912	29	32	24	26	16	127
2	65	171	22,23	17	2932	2772	3640	5598	1440	3800	9702	14942	40	34	31	26	18	149
2	54	161	20,83	16	7330	1733	0	5880	11280	1600	2063	14943	48	44	32	27	15	166
1	80	188	22,63	16	6838	4757	525	2880	3840	4345	6815	15000	39	40	38	31	19	167
2	56	168	19,84	17	2399	8748	1100	2780	2550	11140	1337	15027	27	28	33	20	19	127
1	85	178	26,83	16	6102	7776	1155	133	4380	6595	4191	15166	49	49	42	35	35	210
2	60	179	18,73	16	8519	4788	1020	924	5400	3960	5891	15251	30	41	42	28	9	150
1	59	182	17,81	16	9798	594	0	5022	6480	2400	6534	15414	34	33	29	24	19	139
1	56	166	20,32	16	0	5628	6390	3447	2160	7860	5445	15465	49	49	42	35	35	210
1	40	153	17,09	15	8400	1485	540	5130	5400	5700	4455	15555	36	37	29	25	27	154
2	65	173	21,72	16	5130	4158	1080	5238	3240	1080	11286	15606	47	49	40	35	28	199
1	68	178	21,46	16	4342	3390	1560	6318	0	8680	6930	15610	42	40	33	29	27	171
1	60	174	19,82	16	11718	429	500	2970	7560	500	7557	15617	31	41	30	28	5	135
2	62	184	18,31	16	4165	4998	40	6444	6840	2900	5907	15647	42	37	38	30	25	172
1	60	180	18,52	15	0	0	15750	0	0	15750	0	15750	37	41	30	30	18	156
2	46	159	18,20	16	10030	1793	2880	1116	6180	4540	5099	15819	38	41	31	30	24	164
2	56	169	19,61	15	0	4158	0	11718	7560	0	8316	15876	28	21	26	22	6	103
2	53	162	20,20	15	4755	6363	2450	2346	0	10370	5544	15914	30	31	30	24	19	134
2	70	173	23,39	16	8370	3696	2010	1953	7770	3870	4389	16029	45	45	36	29	30	185
2	46	153	19,65	15	10970	1782	330	3077	5400	3350	7409	16159	24	34	32	25	8	123
1	82	185	23,96	16	2778	10332	0	3090	3420	8820	3960	16200	37	44	6	31	24	142
2	51	155	21,23	17	6570	2376	3290	4010	0	7930	8316	16246	39	31	20	26	13	129

2	62	162	23,62	16	8248	3564	3645	891	5460	4205	6683	16348	35	34	26	26	19	140
2	77	178	24,30	16	6737	5292	1980	2415	2940	7940	5544	16424	16	22	35	20	8	101
2	60	168	21,26	17	12103	462	290	3582	7260	3930	5247	16437	45	47	34	26	26	178
2	54	160	21,09	15	7758	4158	2760	1782	0	6360	10098	16458	26	31	34	23	20	134
1	56	178	17,67	17	1250	5387	255	9576	5070	8675	2723	16468	49	49	39	35	32	204
1	60	177	19,15	16	9810	1782	1890	3039	5850	5490	5181	16521	46	43	32	34	32	187
2	95	166	34,48	16	7614	2970	5090	858	1440	8690	6402	16532	28	27	28	22	18	123
2	45	161	17,36	16	8658	4518	1875	1493	2940	4595	9009	16544	44	47	33	29	33	186
1	68	178	21,46	16	7386	3906	420	4893	6120	7020	3465	16605	26	46	26	26	19	143
1	64	180	19,75	17	8370	3371	1590	3391	3600	7610	5511	16721	47	49	26	33	19	174
1	69	172	23,32	16	7362	2655	2280	4428	5220	8040	3465	16725	44	48	38	33	27	190
1	60	171	20,52	17	6660	4158	3780	2184	4500	5220	7062	16782	49	39	24	35	24	171
1	66	176	21,31	15	12699	1722	90	2303	8280	7230	1304	16814	42	40	37	30	20	169
1	64	178	20,20	17	8580	4932	2160	1158	5400	6480	4950	16830	49	46	29	24	31	179
1	54	168	19,13	15	8724	3267	635	4218	5070	3755	8019	16844	33	38	24	27	18	140
1	60	180	18,52	16	3433	2079	930	10410	10290	3130	3432	16852	43	49	39	34	23	188
1	75	180	23,15	17	8318	99	4140	4380	5760	4940	6237	16937	49	44	28	32	21	174
2	46	160	17,97	17	11970	3210	1820	0	5400	5660	5940	17000	36	39	33	29	20	157
1	75	180	23,15	16	2994	2657	2030	9348	6900	2390	7739	17029	32	44	41	27	14	158
2	48	162	18,29	17	133	9177	125	7623	3000	9405	4653	17058	47	43	41	35	18	184
1	58	180	17,90	17	462	10794	210	5670	3360	7770	6006	17136	33	33	29	25	23	143
1	86	186	24,86	16	3255	3495	4790	5682	4440	8690	4092	17222	33	32	24	26	25	140
1	68	178	21,46	16	4758	11718	55	840	1440	7615	8316	17371	41	30	24	30	9	134
2	52	163	19,57	16	10038	6384	960	0	840	11460	5082	17382	46	41	35	34	24	180
1	54	177	17,24	15	9045	1403	6930	33	2700	9810	4901	17411	38	41	30	24	24	157
2	73	174	24,11	16	11346	3030	1680	1450	4740	8080	4686	17506	43	45	39	35	24	186
1	66	180	20,37	17	7350	2970	3640	3564	780	7240	9504	17524	26	24	18	23	24	115
1	64	183	19,11	17	14238	2772	120	577,5	5040	5160	7508	17708	36	43	30	34	14	157
1	70	185	20,45	16	5313	5019	2750	4697	3840	10210	3729	17779	46	47	36	35	35	199
1	50	173	16,71	15	1211	6045	5260	5285	2520	10760	4521	17801	32	39	28	29	27	155
1	89	192	24,14	17	13090	2600	1300	1015	6480	7020	4505	18005	49	47	34	33	29	192
2	67	176	21,63	16	7399	8022	630	2079	5040	8470	4620	18130	23	25	23	19	19	109
1	72	186	20,81	16	6678	11718	0	0	2520	7560	8316	18396	44	49	37	31	22	183
1	65	173	21,72	16	10044	3237	3840	1287	2880	9060	6468	18408	28	23	40	20	20	131
1	96	184	28,36	17	5398	7593	0	5574	1920	9880	6765	18565	24	26	36	32	23	141
2	60	165	22,04	16	0	4158	6930	7487	4830	6930	6815	18575	49	46	33	29	33	190
1	78	183	23,29	17	0	7479	4090	7038	5400	10930	2277	18607	49	49	33	32	35	198
2	80	155	33,30	17	4138	9342	3780	1386	0	14240	4406	18646	25	23	27	19	18	112
2	49	163	18,44	17	9000	5142	2925	1590	4590	10635	3432	18657	49	41	31	35	23	179
2	62	173	20,72	17	13887	2970	1260	594	7560	6300	4851	18711	28	19	26	17	14	104
2	50	162	19,05	17	12050	1584	2010	3085	7440	5250	6039	18729	41	42	30	34	21	168
1	72	177	22,98	16	13548	693	1260	3306	6270	6300	6237	18807	46	41	27	27	27	168
1	54	175	17,63	16	5205	60	3595	9990	7560	10135	1155	18850	35	44	32	30	21	162
2	54	163	20,32	17	10722	3528	3630	1000	4620	11290	2970	18880	30	31	36	18	10	125
2	46	150	20,44	15	11970	1935	2400	2575	6360	8560	3960	18880	47	48	42	35	31	203
1	55	171	18,81	15	9315	6363	900	2340	5580	7860	5478	18918	44	43	34	33	34	188
2	50	170	17,30	15	6456	2226	7990	2490	2520	10570	6072	19162	10	16	42	18	11	97
2	51	163	19,20	16	10008	1238	360	7560	15120	1900	2145	19165	45	49	41	35	25	195
2	62	176	20,02	16	9558	4158	3715	1782	5400	3715	10098	19213	36	40	37	35	27	175
1	48	165	17,63	15	12690	2415	2700	1428	5640	8280	5313	19233	34	26	25	19	19	123
2	67	170	23,18	15	8570	6362	1440	2862	5400	5270	8564	19234	29	26	36	24	24	139
2	59	173	19,71	17	8150	0	5003	6310	8850	7643	2970	19463	25	20	31	21	21	118
2	51	164	18,96	16	9018	2970	3973	3628	5400	5873	8316	19589	46	46	34	31	31	188
1	68	190	18,84	17	5163	1560	2260	10717	7620	7460	4620	19700	45	46	31	29	27	178
1	62	176	20,02	17	12438	4551	720	2052	5670	7920	6171	19761	47	40	34	30	30	181
2	53	171	18,13	16	9558	264	7380	2853	7560	7380	5115	20055	39	37	36	22	18	152
1	69	192	18,72	16	5790	4050	1020	9198	5400	4560	10098	20058	44	41	39	19	22	165
2	43	164	15,99	16	7638	2928	3150	6492	6390	6690	7128	20208	40	40	35	26	23	164
1	65	175	21,22	17	10890	4158	1425	3744	6480	5025	8712	20217	31	49	27	35	8	150
2	56	165	20,57	17	7290	1938	4928	6102	6480	8168	5610	20258	48	45	38	29	33	193
1	59	175	19,27	16	7530	1155	5220	6490	5940	7360	7095	20395	47	49	37	35	25	193
1	103	177	32,88	18	15318	1485	2580	2070	8640	6180	6633	21453	34	33	24	23	20	134
